# MSK1 mediates BDNF-dependent MeCP2-S421 phosphorylation in postnatal striatal development and psychiatric-relevant behaviours

**DOI:** 10.1038/s41380-026-03630-3

**Published:** 2026-05-18

**Authors:** Natalia Varela-Andrés, Carlos Hernández-del Caño, Alejandro Cebrián-León, Adrián Blanco, Izaskun Los Arcos-López de Pariza, Inés S. Fernández del Campo, Sandra García-Losada, Juan Carlos Arévalo, Manuel Sánchez-Martín, Raquel Bajo-Grañeras, Ricardo Martín, Alberto Sánchez-Aguilera, Miguel A. Merchán, Rubén Deogracias

**Affiliations:** 1 Instituto de Neurociencias de Castilla y León (INCyL). Universidad de Salamanca, Salamanca, Spain; 2 Departamento de Biología Celular y Patología, Facultad de Medicina, Universidad de Salamanca, Salamanca, Spain; 3 Instituto de Investigación Biomédica de Salamanca (IBSAL), Salamanca, Spain; 4 Departamento de Fisiología, Universidad Complutense de Madrid. Instituto Universitario de Investigación en Neuroquímica, Madrid, Spain; 5 Instituto de Investigación Sanitaria del Hospital Clínico San Carlos, Madrid, Spain; 6 Departamento de Medicina, Facultad de Medicina, Universidad de Salamanca, Salamanca, Spain; 7 Servicio de Transgénesis, Plataforma Nucleus, Universidad de Salamanca, Salamanca, Spain

**Keywords:** Neuroscience, Molecular biology, Cell biology, Physiology, Psychiatric disorders

## Abstract

Brain-derived neurotrophic factor (BDNF) is a master regulator of neuronal differentiation and inhibitory circuit maturation in the mammalian brain. Yet, its downstream mediators in distinct neuronal populations remain incompletely defined. Here, we identify mitogen- and stress-activated kinase 1 (MSK1) as a critical mediator of BDNF signalling during postnatal striatal development. MSK1 expression predominates in GABAergic neurons across the cortex and striatum, with region-specific dynamics: MSK1 expression in cortical GABAergic interneurons declines from postnatal day 5 (P5) to day 30 (P30), while expression in striatal GABAergic medium spiny neurons (MSNs) persists into adulthood. Using a novel *Msk1*^*IV*^ KO mouse model, generated by deleting exon IV of *Msk1*, we find that striatal volume and MSN dendritic complexity decrease by P60, without cortical neuron alterations, underscoring MSK1´s striatal-specific role. Mechanistically, MSK1 drives BDNF-induced MeCP2 phosphorylation at serine 421 in MSNs via MAPK/ERK, independently of CaMKII, forming a nuclear complex with MeCP2, thus amplifying MSK1´s role in transcriptional regulation. This MSK1-MeCP2 signalling is also involved in BDNF-dependent and independent morphological developmental processes of cultured striatal neurons. Accordingly, *Msk1*^*IV*^ KO striatum shows dysregulated GABAergic (*Gad1, Gabrg3*) and dopaminergic (*Drd1, Drd2, Drd3*) gene expression, mirroring profiles in MeCP2 deficient models. Behaviourally, *Msk1*^*IV*^ KO mice display hypersociability, impaired nest-building, and increased depressive-like behaviour in the forced swimming test, contributing to striatal circuit dysfunction. These findings link MSK1-mediated molecular disruptions to inhibitory circuit imbalances and behaviours reminiscent of psychiatric disorders, positioning MSK1 as a potential therapeutic target for neurodevelopmental and psychiatric disorders, including those associated with MeCP2 dysfunction.

## Introduction

The development of the mammalian brain is a highly orchestrated process, governed by the interplay of extrinsic signalling cues and intrinsic genetic programs that shape neural circuits and establish precise connectivity patterns [1–6]. Among these signalling cues, neurotrophins —particularly brain-derived neurotrophic factor (BDNF)— are master regulators of neuronal differentiation, survival, and connectivity maturation across brain regions [7–9].

BDNF exerts its effects via tropomyosin-receptor kinase B (TrkB), activating the mitogen-activated protein kinase (MAPK), phosphoinositide 3-kinase (PI3K), and phospholipase C-gamma (PLCγ) pathways [10]. These cascades target nuclear and cytoskeletal elements, driving gene expression and structural remodelling, essential for excitatory and inhibitory neuron maturation. This balance is critical for circuit functionality [11]. Disruptions in this equilibrium are implicated in neurodevelopmental and psychiatric disorders [12]. BDNF’s influence spans development, from neurogenesis [13, 14] to synaptic refinement in adolescence and adulthood [15]. In cortical and hippocampal glutamatergic neurons, BDNF enhances spine formation and synaptic plasticity by upregulating cytoskeletal proteins (MAP2, PSD-95) and immediate-early genes (IEGs; e.g., *Arc*, *c-Fos*) [16, 17]. In GABAergic neurons, including cortical interneurons and striatal medium spiny neurons (MSNs), BDNF regulates dendritic arborization and inhibitory synapse formation [2, 11, 18]. The striatum, where GABAergic MSNs constitute more than 90% of the neuronal population, is a main hub integrating corticostriatal inputs for motor, reward, and habit functions. This brain area relies on BDNF, mainly secreted from corticostriatal projections, for proper MSNs branching and dendritic spine formation [19–21]. In vitro and genetic deletion studies revealed differential BDNF dependency: glutamatergic pyramidal neurons show no changes in their dendrites [22], while GABAergic populations—especially MSNs—exhibit pronounced impairments in dendritic complexity and growth [11, 18–20], suggesting region- and cell-specific signalling modulation.

BDNF also modulates activity-dependent Methyl-CpG-binding protein 2 (MeCP2) phosphorylation [23], which is a key regulator of the development of several brain areas [24, 25], including the striatum [26]. However, the signalling pathways mediating MeCP2 phosphorylation may differ between brain areas: in the cortex and hippocampus, it relies on calcium/calmodulin-dependent protein kinases (CaMKs) [23, 27], whereas in the striatum, the BDNF-dependent pathway controlling MeCP2 activation remains unexplored. Interestingly, MAPK/ERK alterations, linked to anxiety and addiction [28, 29], are also related to autism spectrum disorders (ASDs) and Rett Syndrome [24, 25, 30], neurodevelopmental disorders associated with both BDNF and MeCP2 dysfunction [31, 32].

Among BDNF’s downstream effectors, the nuclear protein mitogen- and stress-activated kinase 1 (MSK1) integrates MAPK/ERK1/2 signalling to regulate gene expression and neuronal maturation [10, 33]. MSK1 is highly enriched in GABAergic-prominent brain regions such as the adult striatum and cerebellum [34, 35]. Functionally, MSK1 mediates BDNF-dependent phosphorylation of histone H3 (H3S10, H3S28) and CREB (S133) in cortical neurons [33, 36, 37], enhancing IEG expression (e.g., *c-Fos*, *Egr1*) [37, 38], critical for synaptic plasticity [39]. Existing full *Msk1* KO mouse models show that in the mature brain, MSK1 facilitates [40] but is not required for [41] hippocampal long-term potentiation. Also, MSK1 regulates circadian control [42], striatal responses in disease states [43, 44], cognition [38] and memory [45]. Despite this, MSK1 developmental role and MSK1-mediated pathways including BDNF-dependent MeCP2 phosphorylation, particularly in GABAergic circuits, remain underexplored.

We hypothesize that MSK1 integrates BDNF signalling in striatal MSN development, acting as a critical mediator of MeCP2-S421 phosphorylation. Using a novel *Msk1* exon IV KO (*Msk1*^IV^ KO) mouse model, we explore its structural, molecular, and behavioural roles. Through this approach, our study aims to delve into the role of MSK1 in striatal MSNs maturation, striatal development and function, and its implications for behavioural deficits associated with neurodevelopmental and psychiatric disorders, such as autism spectrum disorders, schizophrenia and Rett syndrome.

## Materials and methods

Detailed methods are provided in SUPPLEMENTARY INFORMATION.

### Animals

All experiments were approved by the ethical committee of the University of Salamanca and the “Consejería de Agricultura, Ganadería y Desarrollo Rural” of Castilla y León, adhering to European Directive 2010/63/EU and Spanish Royal Decree 53/2013.

### Mouse lines

V*gat-IRES-CRE; Ai9-RCL*^*tdTomato*^ mice were generated by crossing *Vgat-IRES-CRE* (Jackson Laboratory #028862) [46] with *Ai9-RCL*^*tdTomato*^ (Jackson Laboratory #007909) [47] lines. *Msk1*^*IV*^ KO mice were created using CRISPR/Cas9, with specific crRNAs targeting *Msk1* exon IV (Supplementary Table 1). Ribonucleoprotein complexes were microinjected into C57BL/6J zygotes, and offspring were validated by PCR and Sanger sequencing (Supplementary Table 2).

### Histology

Mice were perfused with 4% paraformaldehyde (PFA) and brains were postfixed for 3 h, cryoprotected in 30% sucrose, and sectioned coronally into 40-micron thick slices.

### Immunofluorescence

Sections were permeabilized with 0.25% Triton X-100 in PBS, blocked, and incubated with primary antibodies for 24 h at 4 °C. After washing, slices were incubated with secondary antibodies, mounted on Superfrost slides, and glass-covered using Mowiol/DABCO solution.

#### MSK1 expression during postnatal development

Images of somatosensory cortex, hippocampus, and striatum from postnatal (P) days 5, 10, 15 and 30 (P5, P10, P15 and P30) *Vgat-IRES-CRE; Ai9-RCL*^*tdTomato*^ mice, immunostained for MSK1, NeuN, GFAP, and counterstained with Hoechst 33258, were acquired using a Leica Stellaris SP8 confocal microscope and analysed with Arivis Vision4D.

#### Volumetric assays

Coronal sections were immunostained for DARPP-32, counterstained with Hoechst 33258, and imaged on a Leica Stellaris SP8 confocal microscope. Regions were delimited considering the Allen Brain Reference Atlas, and volumes were estimated using Cavalieri’s Principle in Arivis Vision4D.

### Neuronal morphology

Golgi-Cox-stained neurons [48] from WT and *Msk1*^*IV*^ KO brains were imaged using a Zeiss Axio Observer microscope. Morphology and dendritic spines were analysed with Arivis Vision4D and RECONSTRUCT [49].

E14.5-E16.5 neurons were cultured in supplemented Neurobasal media, transfected with Lipofectamine 2000, and treated with BDNF. Fixed cells were imaged with a Leica Stellaris SP8 confocal microscope and analysed with Arivis Vision4D.

### Spontaneous activity recordings

Acute brain slices were prepared from 8–12-week-old mice perfused with cold NMDG-based solution and stabilized in aCSF. Whole-cell voltage-clamp recordings were acquired to determine sIPSCs and sEPSCs of MSNs.

### MSK1 signalling, interaction assays and quantitative western blot

Corticostriatal brain slices were stabilized in aCSF. Stimulation was performed with modified aCSF (No Mg^2+^, 5 mM Ca^2+^, supplemented with 100 µM bicuculline), as previously described [50, 51].

E14.5-E16.5 neurons were cultured in supplemented Neurobasal media and treated with BDNF, MEK-ERK inhibitor U0126 or CaMKII inhibitor Kn92 (inactive) / Kn93 (active).

Protein lysates were separated on 10% Bis-Tris gels, transferred to PVDF membranes, blocked, and incubated with primary antibodies overnight at 4 °C. Fluorescent secondary antibodies were visualized using an Odyssey XF system, and quantification was performed with Image Studio 6.0.

Endogenous interaction was assessed by co-immunoprecipitation assays in striatal nuclear lysates from P60 WT mice using anti-MSK1 antibodies coupled to protein G magnetic beads.

HEK293-FT nuclear lysates were extracted 72 h post-transfection with MSK1-myc and MeCP2-HA expression plasmids, co-immunoprecipitated using anti-HA magnetic beads and analysed by western blot as previously described.

### Quantitative real-time PCR (qPCR)

cDNA was synthesized from 0.5 µg RNA using RevertAid retrotranscriptase. qPCR was performed with PowerUp SYBR Green Master Mix on a QuantStudio 7, and data were analysed using the 2⁻^ΔΔCt^ method using *Tbp* as reference gene.

### Behavioural tests

All tests were conducted at INCyL animal facilities under controlled light conditions as specified in supplementary information. ANY-maze software was used for tracking and analysis.

### Statistics

Data were analysed using GraphPad Prism 8.

## Results

### Developmental and regional MSK1 expression in GABAergic neurons

**Fig. 1:**
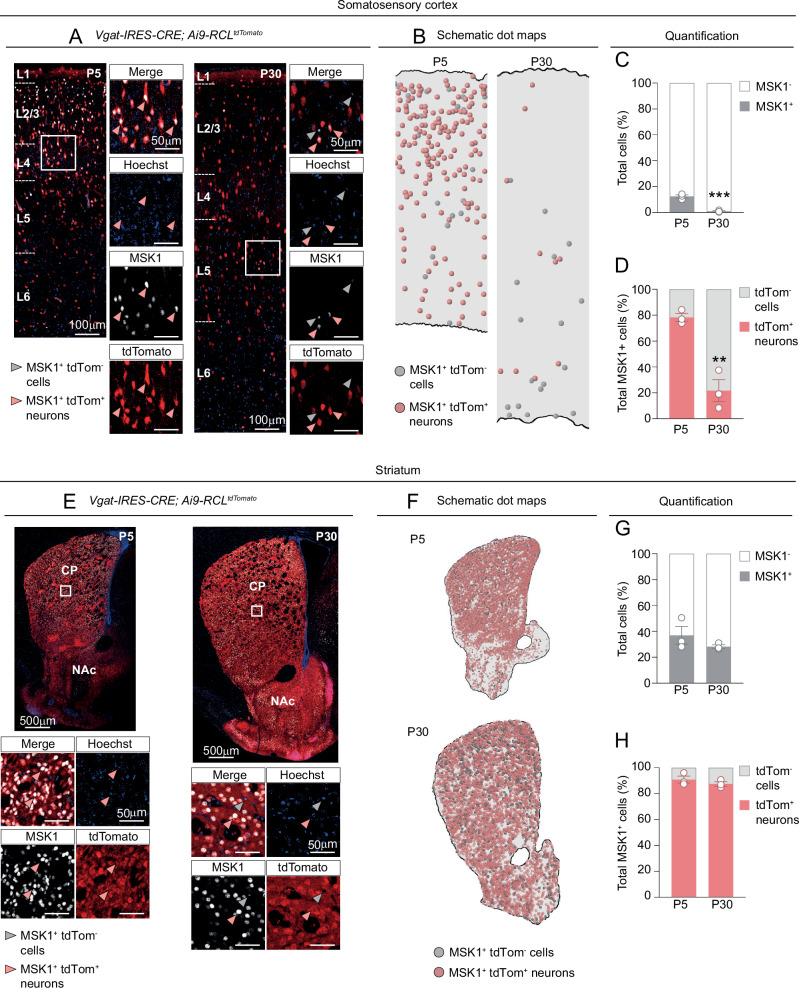
Region-specific MSK1 expression in GABAergic neurons during postnatal mouse development. Analysis of MSK1 expression in *Vgat-IRES-Cre; Ai9-RCL*^*tdTomato*^ mice at postnatal days 5 (P5) and 30 (P30) across somatosensory cortex **A–D** and striatum **E–H**. Each brain region is represented by: representative single-plane confocal images of coronal sections showing MSK1 (grey), tdTomato (red, vast majority of GABAergic neurons), and Hoechst (blue, nuclei) fluorescence, with arrowheads indicating MSK1^+^ non-GABAergic (greyscale) and GABAergic (salmon pink) nuclei **A,** **E**; Schematic dot maps depicting MSK1^+^ cell locations, color-coded as in images **B,** **F**; Quantification of MSK1^+^ cells as a percentage of total cells **C,** **G** and GABAergic MSK1^+^ cells as a percentage of total MSK1^+^ cells **D,** **H** (n = 3 animals per group, 3 sections per region per animal). Scale bars vary by image. Data are presented as mean ± SEM, with each dot representing one animal. Statistical analysis: two-tailed unpaired Student’s t-test (**p < 0.01, ***p < 0.001). See Supplementary Fig. 1–3 for additional characterization and P10 and P15 timepoints with detailed subregion analysis. Abbreviations: P5, postnatal day 5; P30, postnatal day 30; tdTom, tdTomato; L1–L6, cortical layers 1–6; CP, caudate-putamen; NAc, nucleus accumbens.

In the adult murine brain, MSK1 is robustly expressed in the nuclei of striatal MSNs, with lower clustered expression in the cortex and hippocampus [34, 35]. To investigate MSK1 expression in GABAergic neurons during early postnatal development, we used *Vgat-IRES-CRE; Ai9-RCL*^*tdTomato*^ mice, where the vast majority of GABAergic neurons are permanently labelled with tdTomato reporter (Fig. 1), with high specificity and near-complete coverage of inhibitory populations as validated in multiple studies ([52–54]; Allen Brain Atlas connectivity.brain-map.org/transgenic/experiment/100142488) (Fig. 1).

Using immunofluorescence, we quantified MSK1^+^ and MSK1^+^/tdTomato^+^ co-labelled cells in the somatosensory cortex and striatum at P5, P10, P15, and P30 (Fig. 1, Sup. Fig. 1, Sup. Fig. 3). At P5, MSK1^+^ cells accounted for less than 20% of cortical cells (Fig. 1C) and approximately 40% of striatal cells (Fig. 1G). By P30, the proportion of MSK1^+^ cells declined markedly in the cortex relative to P5 (Fig. 1C), whereas striatal levels remained stable (Fig. 1G). Within MSK1^+^ populations (Figs. 1D, H), tdTomato co-labelling was higher than 75% in both regions at P5, dropping to 20% in the cortex by P30 (Fig. 1D), while remaining above 80% in the striatum (Fig. 1H). At P30, most cortical tdTomato^-^/MSK1^+^ cells were NeuN- (Sup. Fig. 1A) and GFAP-negative (Sup. Fig. 1B), indicating they are neither neurons nor astrocytes.

We further analysed MSK1 expression dynamics at P5, P10, P15 and P30 in the somatosensory cortex (across layers) (Sup. Fig. 2A), hippocampal subregions (Sup. Fig. 2B), and striatal domains (Sup. Fig. 3). In the striatum, MSK1 expression across domains (e.g., caudate-putamen, and nucleus accumbens) was quantified as part of the analysis presented in Fig. 1, and Sup. Fig. 3, revealing no significant changes across these timepoints, consistent with persistent MSK1 expression in MSNs into adulthood. In the somatosensory cortex, the proportion of MSK1^+^ cells dropped sharply by P10, stabilizing at P15-P30 across all layers. Co-labelling with tdTomato decreased from P10 onward, with a significant reduction by P30 (Sup. Fig. 2A, panel 3). This decrease occurred earlier in infragranular layers (L5–L6) than in supragranular layers (L1–L4), where higher co-labelling persisted until P15 (Sup. Fig. 2A, panel 4). MSK1^+^ cell distribution, uniform at P5 and P10, shifted to layer 6 by P15-P30 (Sup. Fig. 2A, panel 4). In the hippocampus, MSK1^+^ cells diminished from P10, with significant reductions at P15 and P30 (Sup. Fig. 2B, panel 3), with higher prevalence in CA1 and dentate gyrus (DG) across all stages (Sup. Fig. 2B, panel 4). Co-labelling with tdTomato declined moderately by P10, stabilizing through P30, with higher co-labelling in CA2 at P30 (Sup. Fig. 2B, panel 4). Cells exhibiting the highest MSK1 fluorescence intensity consistently co-localized with tdTomato (Sup. Fig. 2B panel 1 and insets). Our data show that cortical MSK1 is primarily GABAergic during early postnatal stages but shifts to non-GABAergic (tdTomato-negative) cells with maturation, starting in infragranular layers (Sup. Fig. 2A, panels 3 and 4). In the hippocampus, MSK1 expression declines gradually, with co-labelling not exceeding 60%, yet intensely labelled MSK1^+^ cells remain GABAergic (Sup. Fig. 2B, panels 3 and 4). Striatal MSK1 expression showed no significant changes across timepoints (Fig. 1G, H and Sup. Fig. 3) Altogether, these data suggest that MSK1 is predominantly GABAergic across the somatosensory cortex, hippocampus and striatum during early postnatal development, a pattern persisting into adulthood only in the striatum (Fig. 1H and Sup. Fig. 3).

### Spatiotemporal regulation of *Msk1* transcription and protein levels

To investigate developmental changes in *Msk1* expression, we performed qPCR on cDNA from the somatosensory cortex, hippocampus, and striatum of WT mice at P7 and P21. At P7, *Msk1* mRNA levels were higher in the cortex and hippocampus than in the striatum (Sup. Fig. 4A). By P21, cortical and hippocampal *Msk1* mRNA decreased, while striatal levels increased significantly (Sup. Fig. 4A). Western blot analysis confirmed parallel changes in MSK1 protein levels, with reductions in the cortex and hippocampus and an increase in the striatum from P7 to P21 (Sup. Fig. 4B). Analysis of published RNAseq datasheet [6, 55] revealed that *Msk1* expression is predominantly restricted to GABAergic interneurons and oligodendrocytes in the early postnatal (P0–P12) somatosensory cortex (Sup. Fig. 4C), and to MSNs and oligodendrocytes in the adult (P60) striatum (Sup. Fig. 4D).

Altogether, these data reveal region-specific transcriptional and translational regulation of MSK1 during postnatal development.

### Generation and characterization of *Msk1 exon IV knockout (Msk1*^*IV*^*KO)* mice

**Fig. 2:**
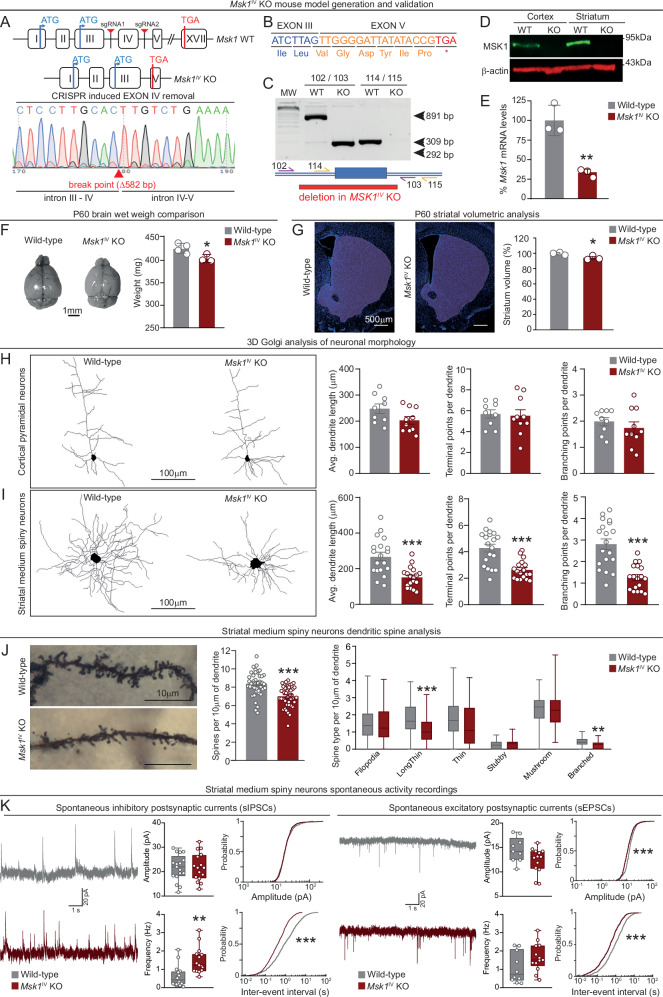
Generation of *Msk1*^*IV*^ KO mice and striatal morphological and functional impairments. **A,** **B** CRISPR/Cas9-mediated targeting of *Msk1* exon IV to introduce a premature stop codon (TGA) in exon V, validated by Sanger sequencing. **C** Genotyping of *Msk1*^*IV*^ KO offspring using dual PCR assays. **D** Western blot analysis of MSK1 protein expression in P60 *Msk1*^*IV*^ KO (KO) mice in cortex and striatum compared to wild type (WT) regions. **E** qPCR analysis of *Msk1* P60 striatal mRNA levels from WT and *Msk1*^*IV*^ KO mice (n = 3 animals per genotype). **F** Brain weight measurement in *Msk1*^*IV*^ KO mice compared to wild type at P60 (n = 3–4 animals per genotype). **G** Representative confocal images of striatal area (left panel) and quantification of striatal volume in WT and *Msk1*^*IV*^ KO mice (n = 3 animals per genotype). **H** Semi-automated tracing of Golgi-Cox-stained cortical pyramidal neurons (left panel) and quantification of neurite length, terminals, and branches in cortical pyramidal neurons (right panel) in WT and *Msk1*^*IV*^ KO mice (n = 9–10 neurons from 3 different animals per genotype; scalebar 100 µm). **I** Semi-automated tracing of Golgi-Cox-stained striatal medium spiny neurons (MSNs) (left panel) and quantification of neurite length, terminals and branches of MSNs (right panel) in WT and *Msk1*^*IV*^ KO animals (n = 18–19 neurons from 3 different animals per genotype; scalebar 100 µm). **J** Representative Golgi-Cox images of dendritic spine density in WT (top left) and *Msk1*^*IV*^ KO (bottom left) MSNs (scalebar 10 µm), and quantification of spine density (middle panel) and spine type distribution (right panel) in *Msk1*^*IV*^ KO versus WT MSNs (n = 45 dendrite segments from 3 animals per genotype, 15 dendrite segments per animal). **K** Spontaneous activity in MSNs. Left: representative traces of spontaneous inhibitory postsynaptic currents (sIPSCs), quantification of mean amplitude (pA) and frequency (Hz), and cumulative distributions of amplitude and inter-event intervals (WT, n = 18 cells from 8 mice; *Msk1*^IV^ KO, n = 15 cells from 7 mice). Right: representative traces of spontaneous excitatory post-synaptic currents (sEPSCs), quantification of mean amplitude (pA) and frequency (Hz), and cumulative distributions of amplitude and inter-event intervals (WT, n = 10 cells from 6 mice; *Msk1*^IV^ KO, n = 13 cells from 7 mice). For sEPSCs (right panel), box plots show mean ± SEM per cell, with no significant genotype differences in mean amplitude or frequency (p > 0.05, Mann-Whitney test). Cumulative probability distributions represent the first 50 individual events per cell (pooled across cells) and were compared using Kolmogorov-Smirnov tests (*p < 0.001), revealing leftward shifts in amplitude and inter-event interval distributions in *Msk1*^*IV*^ KO MSNs. Box plots show median (central line), interquartile range (Q1-Q3; box), and minimum-maximum values (whiskers). (E-J) Data are presented as mean ± SEM, with each dot representing one animal. Statistical analysis: two-tailed unpaired Student’s t-test (*p < 0.05, **p < 0.01, ***p < 0.001); Mann-Whitney U test for median values and Kolmogorov-Smirnov test for cumulative probabilities of individual events (**p < 0.01, ***p < 0.001).

To investigate the role of MSK1 in mouse brain development and adulthood, we generated a novel *Msk1* knockout mouse model by targeting exon IV using CRISPR/Cas9 technology. This approach introduces a premature stop codon (TGA) at the beginning of exon V, resulting in a truncated MSK1 protein (Fig. 2A, B, Sup. Fig. 5A). The exon IV deletion is predicted to generate a short N-terminal peptide (136 aa) lacking all regulatory domains, all kinase activity and the constitutive nuclear localization sequence of MSK1 (Sup. Fig. 5A). However, strong nonsense-mediated decay of the mutant transcript (Fig. 2E) and the requirement for both kinase domains and C-terminal regions for MSK1 function [56, 57] make significant activity or interference by this fragment unlikely. We specifically targeted exon IV, rather than exon I as in previous MSK1 and MSK1/2 KO mouse models, to circumvent potential translation initiation from an alternative in-frame ATG codon located in exon III, which could otherwise serve as a secondary start site. Successful excision of exon IV was verified by Sanger sequencing (Fig. 2A, lower panel), and offspring were genotyped using dual PCR assays (Fig. 2C). The resulting *Msk1*^*IV*^ KO mice are viable and fertile, exhibiting no overt developmental abnormalities by P30, including no differences in size or weight compared to control littermates, nor signs of gross body tremors or other indicators of compromised health. Western blot (Fig. 2D) and immunofluorescence analyses (Sup. Fig. 5B), using antibodies specific to the C-terminal domain of MSK1, confirmed the absence of MSK1 protein in *Msk1*^*IV*^ KO mice. Additionally, *Msk1* transcript levels were significantly reduced compared to controls (Fig. 2E), likely due to nonsense-mediated mRNA decay triggered by the premature stop codon, consistent with previous reports [58]. These findings establish *Msk1*^*IV*^ KO as a robust model for studying MSK1 function, with effective gene silencing and no apparent confounding phenotypes.

### MSK1 deficiency selectively impairs striatal growth and MSNs morphology in vivo

At P60, *Msk1*^*IV*^ KO (KO) brains were significantly lighter than those from wild type (WT) mice (Fig. 2F). Volumetric analysis revealed no differences in the somatosensory cortex (Sup. Fig. 5C) or hippocampus (Sup. Fig. 5D) between *Msk1*^*IV*^ KO and WT mice at P60. However, striatal volume was smaller in *Msk1*^*IV*^ KO mice by P60 compared to controls (Fig. 2G, Sup. Fig. 5E), suggesting a region-specific role for MSK1 in postnatal brain growth. Nevertheless, no differences were observed in the number of cells in each region between genotypes at P60 (Sup. Fig. 5C–E). Given the apparent dependence of striatal development on MSK1, we performed Golgi-Cox staining [48] to assess the morphology of striatal MSNs and pyramidal neurons in the somatosensory cortex, the latter with barely detectable MSK1 expression already at P30 (Fig. 1C). In *Msk1*^*IV*^ KO mice, cortical pyramidal neurons exhibited no changes in neurite length, terminal number per dendrite or branches per dendrite relative to WT (Fig. 2H). In contrast, striatal MSNs from *Msk1*^*IV*^ KO mice displayed a significant reduction in dendritic arbour complexity, with decreased total neurite length and branching (Fig. 2I). Since dendritic spines are primary targets of excitatory cortical afferents, we quantified spine density of secondary dendrites in MSNs and found a significant reduction in *Msk1*^*IV*^ KO mice relative to WT (Fig. 2J), specifically in long thin and branched spines (Fig. 2J, right panel). These findings indicate that MSK1 is necessary for the postnatal growth and morphological maturation of the striatum, particularly its MSNs, but not for other brain areas.

### MSK1 deficiency increases inhibitory synaptic transmission to MSNs

MSNs from *Msk1*^*IV*^ KO mice presented an impaired morphological maturation with a reduced growth of their dendritic arbour. Electrophysiological recordings of MSNs showed a reduction in the membrane capacitance (WT: 36.4 ± 1.4 pF; *Msk1*^*IV*^ KO: 27.7 ± 1.9 pF; *p* = 0.0073), with no statistically significant differences in input resistance (WT: 174.0 ± 9.42 MΩ; *Msk1*^*IV*^ KO: 194.4 ± 18.67 MΩ, *p* = 0.3535) and membrane time constant (WT: 6.30 ± 0.26 ms; *Msk1*^*IV*^ KO: 5.49 ± 0.72 ms, *p* = 0.3182) (All data are expressed as mean ± SEM. Two-tailed unpaired Student’s t-test. WT n = 5 cells, *Msk1*^IV^ KO n = 8 cells). This was also aligned with a reduction in the cell size in MSNs from *Msk1*^*IV*^ KO mice. To evaluate the potential impact of MSK1 deficiency on the synaptic inputs received by MSNs, we recorded spontaneous postsynaptic currents (excitatory, sEPSCs; inhibitory, sIPSCs) from MSNs in 8–12-week-old-mice. We observed that while the amplitude of inhibitory events recorded in these cells was similar for both genotypes, the mean frequency of sIPSCs was significantly higher in MSNs from *Msk1*^*IV*^ KO mice (Fig. 2K, left panel). Regarding sEPSCs recorded in MSNs, we found no statistical differences in the mean amplitude and frequency between WT and *Msk1*^*IV*^ KO mice (Fig. 2K, right panel). Box plots show mean ± SEM per cell, with no significant genotype differences in mean sEPSC amplitude or frequency (p > 0.05, Mann-Whitney test). However, cumulative probability distribution of individual events (first 50 events per cell, pooled across cells) revealed significant leftward shifts in amplitude and inter-event interval distributions in *Msk1*^*IV*^ KO MSNs (*p < 0.001, Kolmogorov-Smirnov test), indicating a higher proportion of smaller and more temporally clustered excitatory events without altering the overall mean per cell (Fig. 2K, right panel). However, cumulative probability distribution of individual events showed a leftward shift of the amplitude and inter-event intervals in MSNs from *Msk1*^*IV*^ KO mice (Fig. 2K, right panel), suggesting an increase in small events and in temporally clustered events in these animals.

These findings indicate that MSK1 is necessary for the postnatal growth and morphological maturation of the striatum, particularly its MSNs, and reveal functional alterations in synaptic transmission consistent with disrupted excitatory/inhibitory balance.

### MSK1 mediates BDNF-dependent MeCP2 phosphorylation at S421 in striatal neurons independently of CaMKII

**Fig. 3:**
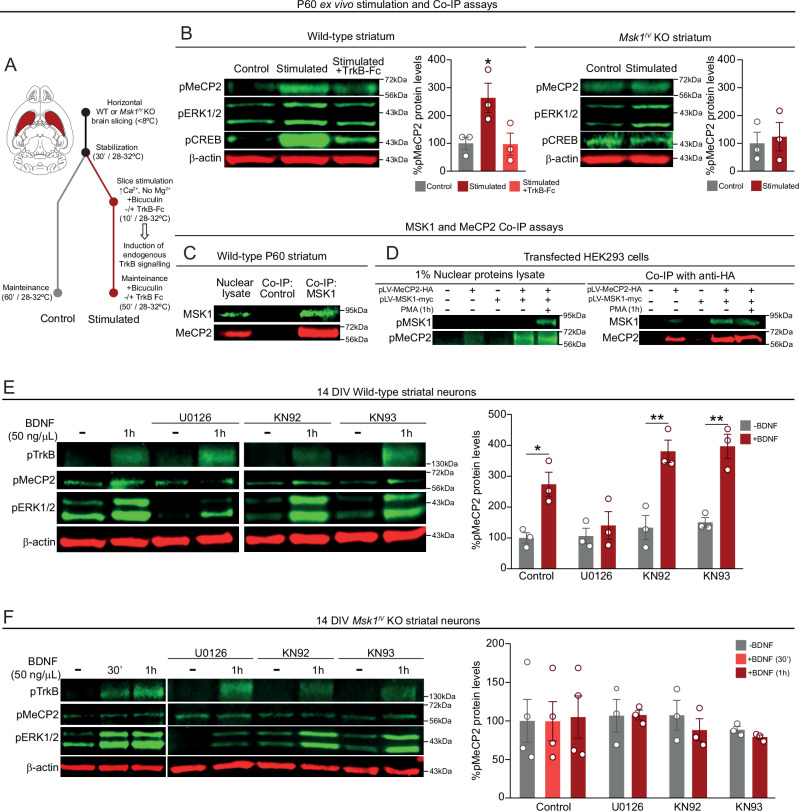
MSK1 mediates BDNF-dependent MeCP2 phosphorylation at Ser421 in striatal neurons via ERK1/2. **A,** **B** Ex vivo induction of endogenous TrkB signalling. **A** Schematic of acute horizontal P60 brain slice preparation and stimulation protocol to induce physiological BDNF release from corticostriatal fibres. **B** Quantitative Western blot analysis of striatal lysates from stimulated P60 WT and *Msk1*^IV^ KO slices, assessing pMeCP2-S421 levels relative to actin in control, stimulated, or stimulated in the presence of TrkB-Fc (2 µg/mL; WT only). n = 3, each n represents a pool of 3–4 slices from the same mouse. Data are presented as mean ± SEM. Statistical analysis: one-way ANOVA with Dunnet´s post hoc test versus control for WT data; two-tailed unpaired Student’s t-test for *Msk1*^IV^ KO data (*p < 0.05). See Supplementary Fig. 6A for pERK1/2 and pCREB-S133 in the same conditions. **C**,**D** MSK1–MeCP2 co-immunoprecipitation assays. **C** Immunoprecipitation of nuclear lysates from P60 WT striatum using control IgG or anti-MSK1 antibody coupled to protein G magnetic beads. **D** Immunoprecipitation of nuclear lysates from HEK293-FT cells co-transfected with MeCP2-HA and MSK1-myc expression plasmids, using anti-HA magnetic beads, to detect MSK1–MeCP2 interaction before and after MSK1 phosphorylation induced by PMA (200 nM, 1 h). **E,** **F** Quantitative Western blot analysis of lysates from 14 DIV wild-type and *Msk1*^*IV*^ KO striatal neurons, assessing pMeCP2-S421 levels relative to actin after treatment with MEK/ERK inhibitor U0126 (100 nM, 30 min), CaMKII inhibitors KN92 (inactive, 10 µM, 1 h) or KN93 (active, 10 µM, 1 h), followed by BDNF stimulation (50 ng/mL, 30 min or 1 h) (n = 3–4, each n representing a pool of 3 wells from the same culture, and each n represent a different neuronal culture). Data are presented as mean ± SEM. Statistical analysis: two-tailed unpaired Student’s t-test (*p < 0.05, **p < 0.01). See Supplementary Fig. 6B–C for pTrkB and pERK1/2 controls in the same conditions.

In cortical and hippocampal neurons, BDNF induces phosphorylation of MeCP2 at serine 421 (pMeCP2-S421) via the CaMKII pathway, independently of MAPK signalling [23, 59]. However, the mechanisms governing this process in striatal neurons remain poorly understood. We found that stimulation of P60 acute horizontal brain slices (Fig. 3A), a protocol known to induce physiological BDNF release and TrkB activation in the striatum [50], induces MeCP2-S421 phosphorylation in the striatum. This effect is blocked by the BDNF scavenger TrkB-Fc in wild-type slices and abolished in slices from *Msk1*^*IV*^ KO mice (Fig. 3B). We also observed that ERK1/2 and the downstream transcription factor CREB follow a similar phosphorylation pattern to MeCP2 upon stimulation, with CREB-S133 phosphorylation disrupted in *Msk1*^*IV*^ KO stimulated slices (Fig. 3B, Supplementary Fig. 6A). These data suggest that MeCP2-S421 phosphorylation may rely on the MAPK/MSK1 signalling pathway in the striatum. To gain insight into this hypothesis, we followed two distinct approaches. The first was to test whether MeCP2 and MSK1, both nuclear-localised proteins, interact within the cell nucleus. We performed co-immunoprecipitation from nuclear lysates of P60 wild-type mouse striatum (Fig. 3C). Endogenous MeCP2 was efficiently co-precipitated with MSK1, demonstrating that the complex exists in vivo in striatal neurons. Specificity was confirmed by lack of signal with control IgG immunoprecipitation. Moreover, we tested this interaction by overexpressing murine HA-MeCP2 and MSK1-myc in a controlled exogenous human system (HEK293-FT cells) followed by co-immunoprecipitation assays using anti-HA magnetic beads in the presence or absence of PMA, an inductor of MSK1 phosphorylation [60]. This experiment revealed that MSK1 and MeCP2 form a complex within the cell nucleus regardless of MSK1 phosphorylation state (Fig. 3D). Our second approach focused on the molecular signalling pathways that drive BDNF-dependent MeCP2-Ser421 phosphorylation in striatal neurons. We observed that BDNF stimulation of cultured striatal neurons from wild-type (WT) mice (E14.5-E16.5 + 14 DIV) increased pMeCP2-S421 levels, alongside pTrkB and pERK1/2 (Fig. 3E, Sup. Fig. 6B), consistent with BDNF activation of TrkB/MAPK/ERK signalling pathway. Notably, pretreatment with U0126 (MEK/ERK inhibitor) abolished BDNF-dependent MeCP2 S421 phosphorylation, whereas pretreatment with KN92 (inactive CaMKII inhibitor) or KN93 (active CaMKII inhibitor) had no effect (Fig. 3E, Sup. Fig. 6B). These data indicate that CaMKII is dispensable for BDNF-dependent MeCP2 S421 phosphorylation in striatal neurons. In contrast, the absence of MSK1 in *Msk1*^*IV*^ KO striatal cultures prevented BDNF-induced MeCP2 S421 phosphorylation, regardless of U0126, KN92 or KN93 pretreatment (Fig. 3F, Sup. Fig. 6B). Collectively, these findings position the ERK/MSK1 signalling pathway as the driver of BDNF-induced MeCP2-S421 phosphorylation in striatal neurons, where MSK1 and MeCP2 associate in the same nuclear protein complex.

### MSK1-MeCP2 signalling regulates arborization of striatal cultured neurons

Having established that postnatal MSK1 expression is enriched in striatal GABAergic neurons (Fig. 1), we next examined whether MSK1 is required for BDNF-driven morphological maturation of striatal neurons in vitro, as their arborization depends on the BDNF-activated MAPK pathway [19]. Immunofluorescence of E14.5 *Vgat-IRES-Cre; Ai9-RCL*^*tdTomato*^ embryonic forebrains confirmed that MSK1 protein is already robustly expressed at this stage in both the cortical plate and ganglionic eminence/striatal primordium, with the vast majority of brightly MSK1-positive cells being tdTomato^+^ GABAergic neurons in both regions (Supplementary Fig. 7A). Thus, embryonic GABAergic neurons destined for cortex or striatum express comparable high levels of MSK1 at the developmental stage used for primary cultures.

**Fig. 4:**
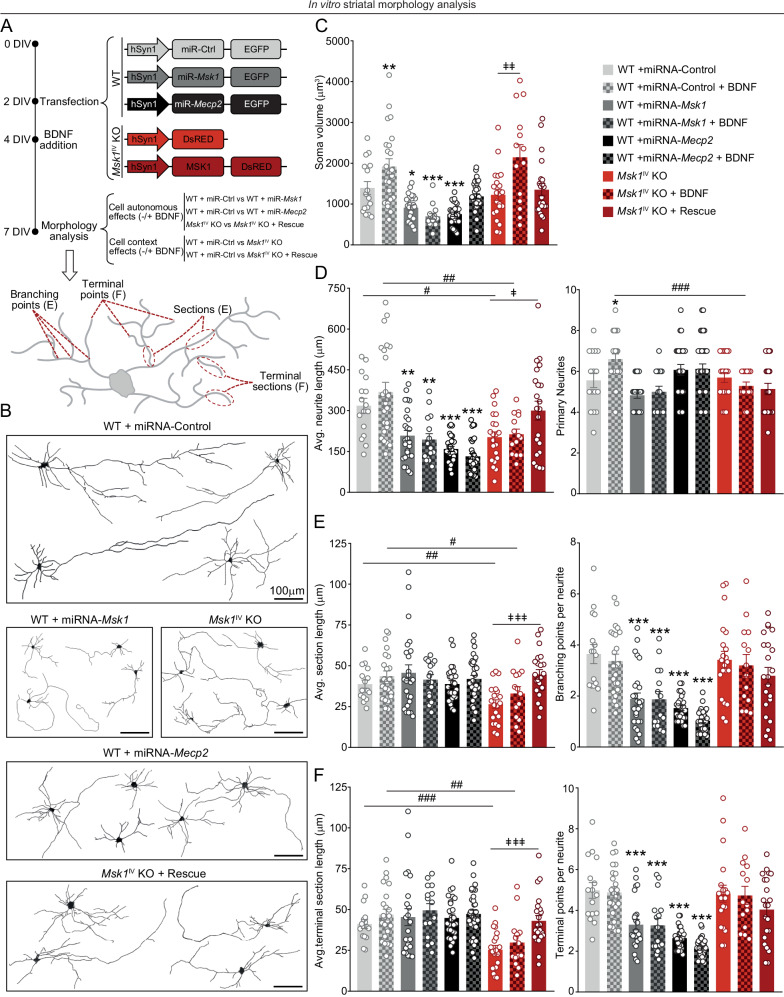
MSK1-MeCP2 signalling regulates striatal neuron arborization in a cell-autonomous and non-cell autonomous manner. **A** Experimental design: striatal neurons from WT and *Msk1*^*IV*^ KO embryos (E14.5-E16.5) were transfected at 2 days in vitro (DIV) with plasmids under the neuronal-specific human synapsin 1 promoter (hSyn1). WT neurons were transfected with hSyn1-miR-Ctrl-EGFP (control miRNA), hSyn1-miR-*Msk1-*EGFP (*Msk1*-targeting miRNAs), or hSyn1-miR-*Mecp2*-EGFP (*Mecp2*-targeting miRNAs). *Msk1*^*IV*^ KO neurons were transfected with hSyn1-DsRED (reporter) or hSyn1-MSK1-DsRED (MSK1 rescue). At DIV 4, cultures were treated with BDNF (50 ng/mL), fixed at DIV 7, and imaged using a Leica Stellaris SP8 confocal microscope. Morphological analysis was performed with Arivis Vision4D. **B** Representative 3D morphological reconstructions of WT striatal neurons after transfection with miRNA-control, miRNA-*Msk1*, or miRNA-*Mecp2*, and *Msk1*^*IV*^ KO striatal neurons after transfection with DsRED expressing plasmids or DsRED + MSK1 rescue plasmid (scale bar 100 µm). **C** Quantification of soma volume in WT and *Msk1*^*IV*^ KO striatal neurons. **D** Quantification of average neurite length and number of primary neurites. **E,** **F** Quantification of arbour complexity including average section length, branching points per neurite, average terminal section length, and terminal points per neurite. Cell-autonomous effects were assessed by intra-genotype one-way ANOVA with Dunnett’s *post hoc* test versus basal condition (WT + miR-Ctrl: *p < 0.05, **p < 0.01, ***p < 0.001; *Msk1*^IV^ KO: ^ǂ^*p* < 0.05, ^ǂǂ^*p* < 0.01, ^ǂǂǂ^*p* < 0.001). Cell-context effects were analysed by two-tailed unpaired Student’s t-test (WT + miR-Ctrl vs. *Msk1*^*IV*^ KO, WT + miR-Ctrl + BDNF vs. *Msk1*^*IV*^ KO + BDNF, WT + miR-Ctrl vs. *Msk1*^*IV*^ KO + Rescue: ^#^*p* < 0.05, ^##^*p* < 0.01, ^###^*p* < 0.001). Data are presented as mean ± SEM. n = 16–33 neurons per condition from 3 different neuronal cultures. See Supplementary Fig. 8 for cortical neuron morphology under the same conditions.

To investigate the roles of MSK1 and its downstream effector MeCP2, we transfected striatal and cortical wild-type primary cultures with either a control microRNA (miRNA-Control), specific miRNAs targeting *Msk1* (miRNA-*Msk1*), or specific miRNAs targeting *Mecp2* (miRNA-*Mecp2*). In parallel, primary cultures from *Msk1*^*IV*^ KO mice were transfected with a DsRed reporter plasmid or an *Msk1* expression plasmid (MSK1-DsRED) to restore MSK1 function. These complementary approaches enabled us to dissect cell-autonomous contributions (miRNA knockdowns) from broader cellular context effects (global KO cultures), and to assess BDNF dependency (Fig. 4A, Sup. Fig. 7).

In WT striatal neurons transfected with a miRNA-Control, BDNF supplementation significantly increased soma volume (Fig. 4C). Cell-autonomous knockdown of either *Msk1 or Mecp2* reduced basal soma volume (Fig. 4C), and BDNF failed to fully restore it in miRNA-*Msk1* but not miRNA-*Mecp2* cultures (Fig. 4C), indicating that intrinsic MSK1 and MeCP2 regulate soma growth with partial overlap. In contrast, in global *Msk1*^*IV*^ KO cultures (lacking MSK1 in all cells), BDNF retained the ability to significantly increase soma volume, and MSK1 re-expression in a subset of neurons did not alter this response in the absence of BDNF (Fig. 4C). This reveals non-cell-autonomous compensation that partially bypasses the intrinsic requirement for MSK1 in somatic growth when the entire microenvironment is MSK1-deficient. BDNF treatment of control-transfected wild-type striatal neurons significantly increased total neurite length (miRNA-Control: 1658 ± 187.9 µm vs. miRNA-Control + BDNF: 2473 ± 249.3 µm; mean ± SEM; *p* = 0.0151, two-tailed unpaired Student’s t-test), consistent with prior reports [18], and primary neurite number (Fig. 4D). Average neurite length was unaffected by BDNF in control conditions but was reduced by both cell-autonomous *Msk1*/*Mecp2* knockdown and global KO, with rescue upon MSK1 re-expression (Fig. 4D, left panel). The BDNF-induced increase in primary neurite number was abolished in global KO and by cell-autonomous *Msk1* knockdown, with no rescue after MSK1 over-expression (Fig. 4D right panel). *Mecp2* knockdown phenocopied MSK1 loss for both average neurite length and BDNF-induced increase of number of primary neurites, supporting MeCP2 as a downstream effector of MSK1 in these processes. Given that distinct neurite segments are governed by an interplay of intracellular and extracellular mechanisms [6], we examined branching complexity (Fig. 4E,F). Average section length (Fig. 4E, left panel) and average terminal section length (Fig. 4F, left panel) were selectively reduced in *Msk1*^*IV*^ KO striatal cultures (BDNF-independent), and rescue by MSK1 re-expression, indicating a predominant microenvironment-dependent role for MSK1 in neurite segment elongation. In contrast, the number of branching points (Fig. 4E, right panel) and terminal points per neurite (Fig. 4F, right panel) were impaired by both cell-autonomous *Msk1* and *Mecp2* knockdown, with *Mecp2* loss phenocopying *Msk1* deficiency, highlighting intrinsic MSK1-MeCP2 signalling in branching frequency. These differences highlight two distinct mechanisms that cause striatal neurite impairments upon *Msk1* loss, in which cell-autonomous disruption of MSK1-MeCP2 signalling affects the number of times a neurite branches, whereas global *Msk1* absence impairs the extension between branching points and from them to the terminal regions.

Contrary, cell-autonomous MSK1 deletion in cortical neurons did not exert any effects on neuronal morphology, whereas global loss in *Msk1*^IV^ KO cultures caused abnormal BDNF-dependent morphological responses (Sup. Fig. 8). *Mecp2* knockdown impaired cortical morphology as previously reported [61, 62], but *Msk1* disruption did not phenocopy *Mecp2* loss, consistent with region-specific MSK1-MeCP2 functional dependence.

Collectively, these complementary genetic approaches demonstrate that MSK1-MeCP2 signalling regulates both BDNF-dependent and -independent aspects of striatal neuron arborization. Primary neurite outgrowth, total neurite length, and branching frequency are strictly cell-autonomous and require intrinsic MSK1–MeCP2 signalling, whereas soma growth and neurite segment elongation show partial non-cell-autonomous compensation in global knockout conditions.

### MSK1 regulates the expression of genes related to the GABA and dopamine system

**Fig. 5:**
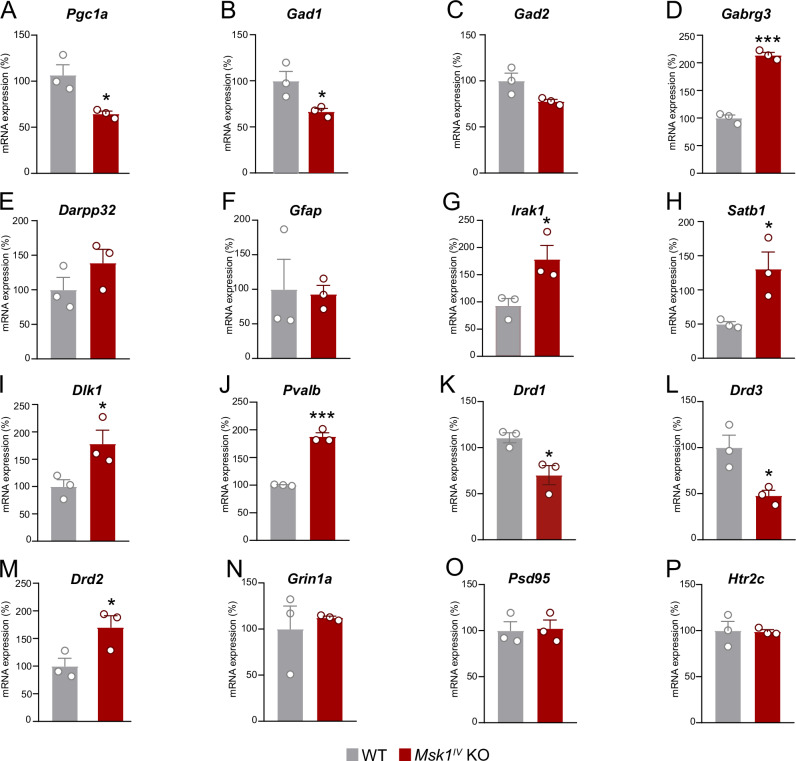
MSK1 deficiency dysregulates striatal gene expression. qPCR analysis of striatal cDNA from P60 wild-type (WT) and *Msk1*^*IV*^ KO mice, showing mRNA levels of: **A** *Pgc1α* (*Ppargc1a*; PPARG coactivator 1 alpha), **B** *Gad1* (glutamate decarboxylase 1), **C** *Gad*2 (glutamate decarboxylase 2), **D** *Gabrg3* (GABA-A receptor subunit gamma 3), **E** *Ppp1r1b* (DARPP-32), **F** *Gfap* (glial fibrillary acidic protein), **G** *Irak1* (interleukin 1 receptor-associated kinase 1), **H** *Satb1* (special AT-rich sequence-binding protein 1), **I** *Dlk1* (delta-like non-canonical Notch ligand 1), **J** *Pvalb* (parvalbumin), **K** *Drd1* (dopamine receptor 1), **L** *Drd3* (dopamine receptor 3), **M** *Drd2* (dopamine receptor 2), **N** *Grin1a* (NMDA receptor subunit 1a), **O** *Psd95* (postsynaptic density 95), and **P** *Htr2c* (5-hydroxytryptamine receptor 2C). Data are presented as mean ± SEM. Statistical analysis: two-tailed unpaired Student’s t-test (*p < 0.05, ***p < 0.001). n = 3 animals per genotype.

To further explore the transcriptional role of MSK1 in the striatum and its contribution to regulating MeCP2-dependent gene expression, we conducted qPCR on cDNA from the striatum of P60 *Msk1*^*IV*^ KO mice and WT littermates (Fig. 5). First, we analysed *Pgc1α* (*Ppargc1a*, peroxisome proliferator-activated receptor gamma coactivator 1-alpha), a gene previously shown to depend on MSK1 [44], and found a significant reduction in *Msk1*^*IV*^ *KO* striatum (Fig. 5A). Our analysis on GABA-related genes, revealed a significant reduction in glutamate decarboxylase 1 (*Gad1*) but not glutamate decarboxylase 2 (*Gad2*), encoding the GABA-synthesizing enzymes GAD67 and GAD65 respectively, alongside a marked upregulation of GABA-A receptor subunit gamma 3 (*Gabrg3*) in *Msk1*^*IV*^ KO mice compared to WT controls (Fig. 5B–D). In contrast, mRNA levels of the MSN-marker gene *Darpp-32* (dopamine- and cAMP-regulated phosphoprotein, 32 kDa) (Fig. 5E) and the astrocytic marker *Gfap* (glial fibrillary acidic protein) (Fig. 5F) remained unchanged. The absence of MSK1 also dysregulated other striatal function-related genes, increasing *Irak1* (interleukin-1 receptor-associated kinase 1), *Satb1* (special AT-rich sequence-binding protein 1), *Dlk1* (delta-like non-canonical Notch ligand 1), and *Pvalb* (parvalbumin) expression (Fig. 5G–J). Notably, *Irak1*, *Satb1*, and *Dlk1* show the same upregulation in *Mecp2-*deficient models [63], consistent with impaired BDNF-driven MeCP2-S421 phosphorylation contributing to a subset of the observed transcriptional alterations [63]. Extending our analysis to dopamine receptor genes, known MeCP2 targets, we found reduced mRNA levels of *Drd1* (Fig. 5K) and *Drd3* (Fig. 5L), encoding dopamine receptors Drd1 and Drd3, respectively, whereas *Drd2* (dopamine receptor Drd2) expression was significantly elevated in the *Msk1*^*IV*^ *KO* striatum (Fig. 5M). These dopamine receptor alterations align with prior observations in *MeCP2* knockout mice [63, 64]. Conversely, no significant changes were detected in *Grin1* (glutamate ionotropic receptor NMDA type subunit 1a) (Fig. 5N), *Psd95* (postsynaptic density protein 95) (Fig. 5O), or *Htr2c* (5-hydroxytryptamine receptor 2C) (Fig. 5P). Altogether, these data demonstrate that MSK1 regulates transcription of genes critical for GABAergic and dopaminergic network function in the striatum, contributing to molecular profiles associated with MeCP2 dysfunction.

### MSK1 deficiency alters behaviours relevant to psychiatric disorders

**Fig. 6:**
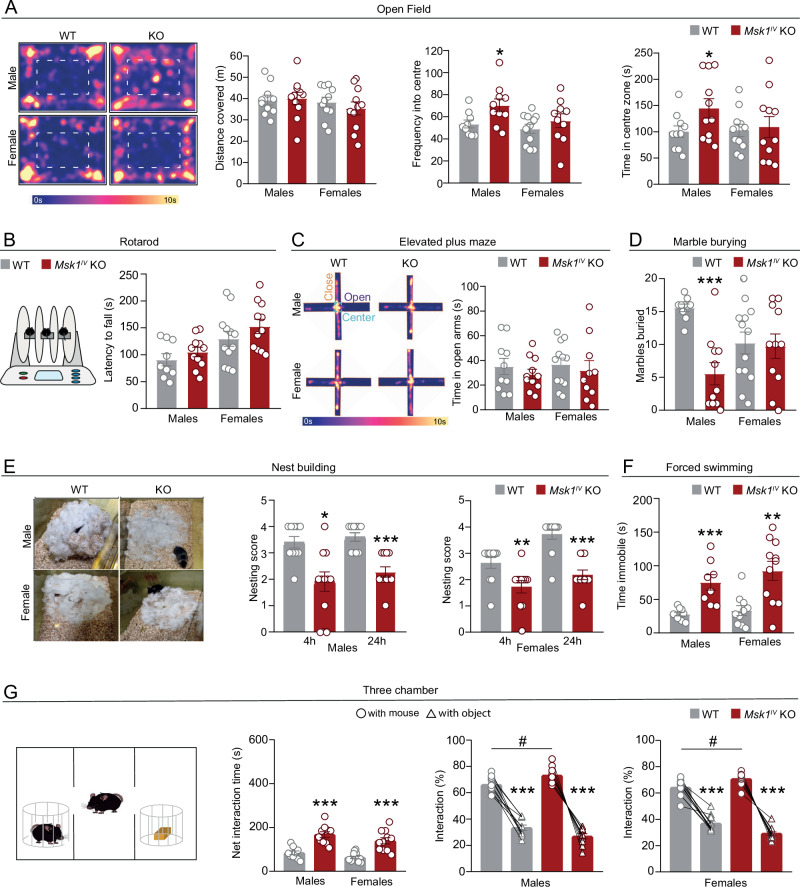
MSK1 deficiency alters behaviours relevant to psychiatric disorders. Behavioural analysis of P60 wild-type (WT) and *Msk1*^*IV*^ KO mice. **A** Open field test: heatmaps (colour scale: 0–10 s), distance travelled (m), centre zone entries, and time in centre (s). **B** Rotarod test: apparatus schematic and latency to fall (s). **C** Elevated plus maze: heatmaps (colour scale: 0–10 s) and time in open arms (s). **D** Marble-burying test: number of marbles buried. **E** Nest-building test: representative images at 24 h and nesting scores at 4 and 24 h. **F** Forced swimming test: immobility time (s). **G** Three-chamber social test: arena schematic, net interaction time with conspecific (s), and social preference (% interaction = % time sniffing mouse / total sniffing time). n = 9–13 animals per column. Data are presented as mean ± SEM. Statistical analysis: For net interaction time (s) (left graph): two-tailed unpaired Student’s t-test (*p < 0.05, **p < 0.01, ***p < 0.001); For males interaction (%) (middle graph): Two-way repeated measures ANOVA with Sidak´s multiple comparisons test: ^#^*p* = 0.0263, ***p < 0.0001; For females interaction (%) (right graph): Two-way repeated measures ANOVA with Sidak´s multiple comparisons test ^#^*p* = 0.0134, ***p < 0.0001.

To investigate the behavioural consequences of MSK1 deficiency, we assessed locomotor activity, social responses, and affective behaviours in P60 *Msk1*^*IV*^ KO mice of both sexes and WT littermates as controls. Locomotor function, evaluated via the open field test (Fig. 6A) and accelerating rotarod assay (Fig. 6B), showed no significant differences in distance travelled or latency to fall between *Msk1*^*IV*^ KO mice versus WT littermate controls. Anxiety-like responses were examined using the open field test (Fig. 6A) and elevated plus maze (Fig. 6C). In the open field, *Msk1*^*IV*^ KO males, but not females, entered the centre zone more frequently and spent significantly more time there compared to WT controls (Fig. 6A), suggesting reduced anxiety-like behaviour in males. However, both sexes showed no differences in time spent in the open arms of the elevated plus maze (Fig. 6C), indicating context-specific anxiety responses. Next, we investigated innate and specific social behaviour. In the marble-burying test, *Msk1*^*IV*^ KO males, but not females, buried significantly fewer marbles than WT controls (Fig. 6D), suggesting a male-specific reduction in repetitive or anxiety-driven behaviour. In the nest-building test *Msk1*^*IV*^ KO males and females exhibited significantly impaired nest-building performance at both 4- and 24- h timepoints compared to WT controls (Fig. 6E). The forced swimming test (FST) revealed that *Msk1*^*IV*^ KO mice of both sexes remained immobile for significantly longer durations than WT littermates (Fig. 6F), indicative of increased depressive-like behaviour. Finally, in the three-chamber social test, both WT and *Msk1*^*IV*^ KO mice showed clear preference for the conspecific over the object (Fig. 6G). However, *Msk1*^*IV*^ KO mice displayed significantly higher preference (% time sniffing mouse / total sniffing time) and absolute interaction time with the conspecific than WT littermates (Fig. 6G), confirming the hypersociability phenotype. Collectively, these findings demonstrate that MSK1 deficiency alters social, repetitive behaviours and increases depressive-like behaviours in adult mice.

## Discussion

Our study positions MSK1 as a critical mediator of BDNF-MeCP2 signalling in postnatal striatal development. We demonstrate that MSK1 exhibits a region-specific-developmental trajectory: enriched in GABAergic neurons across the cortex and striatum at early postnatal stages (P5 to P15), its expression diminishes in cortical populations by P30, persisting robustly in MSNs into adulthood (Fig. 1, Sup. Figs. 1–3). This spatiotemporal pattern aligns with BDNF’s critical role in inhibitory neuronal maturation and circuit formation [2, 18, 21] and suggests MSK1 acts as a key mediator of neurotrophic effects in the development of inhibitory populations, particularly in the striatum. Previously reported RNAseq data [6] show that *Msk1* expression is highest in PV^+^ chandelier cells, with minimal levels in pyramidal neurons at P12 (Sup. Fig. 4C). Interestingly, at P30 cortical MSK1^+^ cells are NeuN-/GFAP-, potentially oligodendrocytes, as suggested by high *Msk1* mRNA levels [6, 55] and morphological features resembling satellite oligodendrocytes [65] (Sup. Fig. 1A), though this requires marker confirmation. To explore MSK1’s functional role, we generated the *Msk1*^*IV*^ KO model (Fig. 2, Sup. Fig. 4A), designed to preclude alternative translation from exon III. Our model reveals selective striatal deficits absent in prior MSK1 models targeting exon I [36]. The exon IV deletion predicts a short N-terminal peptide (136 aa) lacking all kinase and regulatory domains (Sup. Fig. 4A). However, strong nonsense-mediated decay of the mutant transcript (Fig. 2E) and the requirement for both kinase domains and C-terminal regions for MSK1 function [56, 57] make significant activity or interference by this fragment unlikely. Unlike previous MSK1 models targeting exon I and MSK1/2 double KOs, which exhibit no gross CNS developmental defects [66], our model shows reduced brain weight and striatal volume by P60 (Fig. 2F–G, Sup. Fig. 5E), with MSNs displaying impaired dendritic complexity, spine density, and enhanced inhibitory synaptic drive (Fig. 2I–K), while cortical morphology remains intact (Fig. 2H). These structural and functional alterations mirror those in CNS conditional BDNF knockout mice [18], where striatal MSN dendritic complexity is reduced, while glutamatergic pyramidal neuron morphology remains intact in both models. Prior MSK1/2 KO mouse models show hippocampal deficits only under stress, such as pilocarpine-induced seizures [67] or cerebral ischemia [68]. These studies suggest that both MSK1 and MSK2 support neuronal progenitor proliferation and maturation in the subgranular zone (SGZ) under pathological conditions, roles less evident in our unstressed *Msk1*^*IV*^ KO mice, where hippocampal integrity may reflect compensatory RSK activity [69, 70], pending further study. In the adult striatum, MSK1 regulates dopamine-induced histone H3 phosphorylation and gene expression (e.g., *c-Fos*), modulating reward responses [71], a function our developmental deficits may prefigure. Moreover, our in vitro studies correlate with central findings on BDNF roles, where GABAergic neurons and striatal MSNs show pronounced dendritic impairments compared to lack of effects in pyramidal neurons [11, 18, 20, 22]. Indeed, we found that in striatal cultures MSK1 mediates BDNF-driven increases in soma size, neurite elongation, and branching, effects impaired or partially compensated in *Msk1*^*IV*^ KO cultures (Fig. 4, Sup. Fig. 7), whereas cortical neurons exhibit compensatory BDNF responses (Sup. Fig. 8), highlighting MSK1-dependent cell-type-specific signalling dynamics. The differences observed between cell-autonomous miRNA knockdown and global *Msk1*^*IV*^ KO cultures (Fig. 4) are more consistently explained by non-cell-autonomous compensatory mechanisms in the full knockout microenvironment (e.g., altered paracrine signalling, glial contributions, or network adaptations) rather than interference by a hypothetical truncated or unstable MSK1 peptide. This interpretation is supported by: (i) strong nonsense-mediated decay, which substantially reduces mutant transcript levels (Fig. 2E); (ii) the predicted short N-terminal fragment (136 aminoacids) lacking all kinase domains, regulatory regions, and the constitutive nuclear localization sequence, rendering significant functional activity or dominant-negative interference unlikely; and (iii) absence of dominant-negative effects in prior MSK1 knockout models or in our functional assays. The in vitro assays revealed distinct mechanisms underlying striatal neurite impairments in *Msk1* loss. Cell-autonomous knockdown of *Msk1* or *Mecp2* (via miRNA) primarily reduced branching frequency (number of branching/terminal points per neurite) and BDNF responsiveness in primary neurite number, consistent with intrinsic MSK1-MeCP2 nuclear signalling regulating transcriptional programs for dendritic branching (e.g., via CREB/H3 phosphorylation and IEGs). In contrast, global *Msk1*^IV^ KO cultures showed preserved branching points but impaired neurite segment elongation and terminal extension (BDNF-independent), suggesting non-cell-autonomous compensation or microenvironmental factors (e.g., altered secreted cues from neighbouring KO cells, glial support, or network activity) partially rescue branching but not full arbor extension. This dichotomy aligns with literature on cell-autonomous vs. non-cell-autonomous effects in neuronal morphology, where intrinsic signalling governs local branching decisions, while global deletion allows compensatory adaptations via paracrine/glial signals or network remodelling. Regarding the apparent discrepancy with in vivo Golgi-Cox data (reduced branching points and terminal points per dendrite in *Msk1*^*IV*^ KO MSNs), several factors may contribute: (i) in vivo MSNs integrate over longer developmental periods with corticostriatal inputs and experience-dependent refinement absent in vitro; (ii) in vitro cultures (E14.5 – E16.5 + 14 DIV) capture early arborization, while in vivo P60 reflects mature deficits accumulating postnatally; (iii) local compensatory mechanisms (that are also present in isolated cultures) may be overridden or suppressed only in the intact circuit in vivo by circuit-level feedback, excessive corticostriatal afferent drive, or activity-dependent pruning, resulting in more pronounced long-term deficits in branching complexity. These differences highlight complementary insights from both approaches: in vitro dissects cell-intrinsic mechanisms, while in vivo reveals integrated, long-term consequences in the intact circuit.

A central finding is MSK1’s mediation of BDNF-dependent MeCP2 phosphorylation at S421 in striatal neurons via the MAPK/ERK pathway, not CaMKII, unlike cortical and hippocampal neurons [23, 27]. This striatal-specific MAPK/ERK/MSK1-MeCP2 axis is supported by MSK1-MeCP2 nuclear complex formation (Fig. 3C,D), physiological BDNF-induced MeCP2-S421 phosphorylation requiring MSK1 (Fig. 3A,B), and MeCP2 knockdown phenocopying MSK1 deficiency in striatal (but not cortical) morphology and BDNF responsiveness (Fig. 4). Striatal-specific qPCR data reinforce this axis (Fig. 5), showing altered expression of genes relevant to GABAergic and dopaminergic functionality, synaptic formation, and MSN maturation. Reduced *Gad1* expression (encoding GAD67) (Fig. 5B) and increased *Gabrg3* (gamma-aminobutyric acid type A receptor subunit gamma 3) (Fig. 5D) suggest impaired GABA synthesis and compensatory inhibitory tone, paralleling MeCP2-deficient mouse models [63, 72]. Dysregulated dopamine receptors (decreased *Drd1* and *Drd3*, increased *Drd2*; Fig. 5K–M) mirror schizophrenia-like imbalances, as nigrostriatal dopaminergic signalling dysfunction stands as a key feature [73], together with identified *DRD1* [74] and *DRD2* [75] expression changes. DRD2 alterations in the striatum have also been reported in Rett Syndrome patients [76], alongside with striatal morphological alterations described in MeCP2-null mice [77]. Additional alterations in *Irak1*, *Satb1*, *Dlk1*, *Pvalb* (Fig. 5G–J) and *Pgc1α* (Fig. 5A)—genes tied to MeCP2-mediated neuronal maturation [63, 78] and MSK1 activity [44]— link MSK1 to BDNF/MeCP2-mediated networks. Notably, *Irak1*, *Satb1*, and *Dlk1* show the same upregulation in MeCP2-deficient models [63], consistent with impaired BDNF-driven MeCP2-S421 phosphorylation contributing to a subset of the observed transcriptional alterations. Unchanged *Grin1*, *Psd95*, and *5Htr2c* (Fig. 5N–P) expression indicates specificity to GABA/dopamine systems over glutamatergic or serotonergic inputs, aligning with the selective MSN vulnerability. These molecular disruptions directly correlate with the observed MSN morphological deficits and functional alterations in synaptic transmission. Reduced dendritic arborization and spine density (Fig. 2I–J) reflect impaired corticostriatal synaptic integration [18]. In addition, decreased membrane capacitance and increased frequency of spontaneous inhibitory postsynaptic currents (sIPSC; Fig. 2K) indicate enhanced inhibitory drive onto MSNs, consistent with upregulation of *Pvalb* (Fig. 5J) and *Gabrg3* (Fig. 5D) expression. The reduced spine density suggests a loss of excitatory afferents, potentially compensated by strengthened inhibitory connections. Although mean amplitude and frequency of spontaneous excitatory postsynaptic currents (sEPSCs) showed no differences, cumulative distributions revealed a shift toward smaller amplitudes and shorter inter-event intervals (Fig. 2K), suggesting increased small and temporally clustered excitatory events. Although no glutamatergic genes (e.g., *Grin1*, *Psd95*) were significantly altered in our qPCR analysis, the shift toward smaller and more clustered sEPSCs in *Msk1*^*IV*^ KO MSNs likely arises from postsynaptic morphological deficits, including reduced dendritic arborization and spine density (Fig. 2I,J). These structural changes can trigger presynaptic compensatory mechanisms at remaining corticostriatal synapses, such as increased release probability or event clustering without requiring widespread transcriptional dysregulation of glutamatergic components. These data, together with the morphological alterations in *Msk1*^*IV*^ KO MSN dendritic morphology (Fig. 2I–J) and the developmental deficits observed in vitro (Fig. 4), indicate disrupted excitatory/inhibitory balance and altered striatal circuit configuration in *Msk1*^*IV*^ KO mice, a hallmark of neurodevelopmental disorders [12, 87]. Reduced *Gad1* expression (Fig. 5B) may impair GABA synthesis, while the increased sIPSC frequency and upregulated *Gabrg3*/*Pvalb* suggest compensatory strengthening of inhibitory tone. Dopamine receptor imbalances (decreased *Drd1*/*Drd3*, increased *Drd2*; Fig. 5K–M) could further disrupt circuit function, altogether limiting MSN maturation [79, 80]. In addition, *Drd1*/*Drd3* downregulation and *Drd2* upregulation could reflect pathway-specific dysregulation rather than a shift in MSN subtype proportions, as the latter would likely produce severe motor deficits not observed in *Msk1*^*IV*^ KO mice (normal locomotion and rotarod performance; Fig. 6A–B). Both direct and indirect pathway MSNs express high *Msk1* levels in adult striatum (Sup. Fig. 4D), supporting transcriptional effects within existing populations. These synaptic and structural deficits mirror those observed in BDNF conditional knockout models [18, 20, 22], underscoring MSK1’s role in translating neurotrophic cues into MSN maturation.

Behaviourally, *Msk1*^*IV*^ KO mice exhibited hypersociability, severely impaired nest building, reduced male marble burying, and increased FST immobility, with intact locomotion (as demonstrated in open field and rotarod) and no consistent anxiety-related features in the open field and elevated plus maze tests (Fig. 6A–C). These phenotypes are consistent with striatal circuit dysfunction. The behavioural domains affected are well-established to depend, at least in part, on normal striatal function, and the selective anatomical pathology in striatal volume and MSN morphology, in the absence of comparable cortical changes, supports the striatum as a major contributor to these alterations. Although contributions from other MSK1-expressing regions cannot be formally excluded in this constitutive knockout, the behavioural profile closely matches phenotypes reported in more restricted striatal genetic models and aligns with striatal MSN deficits disrupting cortico-striatal circuits [81, 82]. Reduced marble burying (Fig. 6D), a mark of repetitive, compulsive-like behaviour [83, 84], is linked to striatal development [85] and prefrontal-striatal connectivity misregulation [86], also present in schizophrenia *Disc1*^*∆2-3/∆2-3*^ models [87]; it likely stems from MSN dendritic impairments, disrupting top-down control. Increased *Drd2* expression may drive psychosis-like symptoms, while reduced MSN connectivity contributes to social deficits. Nest-building deficits are a sensitive readout of basal ganglia dysfunction, as evidenced by their direct correlation with striatal damage in models of nigrostriatal toxicity [88] and striatal infarct following stroke [89]. Similar to *Erbb-4*-deleted and *Glud1*-deleted mice [90], established schizophrenia-like models, *Msk1*^IV^ KO mice display disrupted nest-building behaviour (Fig. 6E). This reflects self-neglect attitudes, characteristic in schizophrenia patients [91]. Increased immobility in the forced swimming test has been repeatedly associated with striatal dysfunction in models of neuropsychiatric disorders [82]. FST immobility in the *Msk1*^IV^ KO mice indicates increased depressive-like behaviour [92], which is also linked to dopamine dysregulation [93], suggesting negative symptoms akin to several psychiatric disorders. Hypersociability traits in the three-chamber test (Fig. 6G), resembling Dlx5/6-MeCP2^-/y^ mice [72], may arise from MeCP2-dependent dopamine dysregulation in the striatum [64], as well as dopamine deficits in other areas like the ventral tegmental area [94] or the prefrontal cortex [95], two main striatal BDNF sources [96, 97]. While elevated immobility in the FST indicates increased depressive-like behaviour, consistent with striatal circuit alterations and dopamine dysregulation, we acknowledge that this relies on a single paradigm. Future studies using complementary tests (e.g., tail suspension test or sucrose preference) would further validate this phenotype. Notably, *Msk1*^*IV*^ KO mice do not display generalized anxiety-like behavior (normal performance in elevated plus maze and open-field center time) or gross locomotor deficits (normal spontaneous locomotion and rotarod performance; Fig. 6), further arguing against widespread disruption of non-striatal circuits. Thus, while extra-striatal regions may contribute, the selective anatomical pathology in the striatum together with a behavioral profile that closely recapitulates phenotypes linked to striatal dysfunction strongly support the conclusion that the striatum is a major affected region and a key contributor to the behavioral deficits observed in *Msk1*^*IV*^ KO mice. Our battery — covering locomotion, sociability, anxiety, and depression — robustly captures these traits, with sex-specific schizophrenia-like differences [98].

Our findings establish MSK1 as a critical regulator of striatal circuits underlying psychiatric disorders, particularly schizophrenia and autism spectrum disorders (ASD). The observed reductions in *Gad1* expression, enhanced inhibitory drive, dysregulated dopamine receptors expression (decreased *Drd1*/*Drd3*, increased *Drd2*) mirror striatal imbalances in these disorders, with DRD2 receptor hyperactivity contributing to psychosis-like symptoms [73–76]. Morphological deficits in MSNs, including reduced dendritic arborization and spine density, impair corticostriatal connectivity, potentially underlying the hypersociability, depressive-like behaviours, and impaired nest building observed in *Msk1*^*IV*^ KO mice. These phenotypes resemble negative and social symptoms displayed in schizophrenia and ASD patients [91–93], and parallel MeCP2-deficient models, where striatal dopamine dysregulation drives ASD-like traits [63, 64]. By mediating BDNF-dependent MeCP2 phosphorylation via the MAPK/ERK1/2, MSK1 integrates neurotrophic signalling into transcriptional networks governing GABAergic and dopaminergic function. The *Msk1*^*IV*^ KO model thus links molecular, structural, functional and behavioural outcomes, positioning MSK1 as a potential therapeutic target within MAPK signalling pathways for restoring circuit balance.

Future studies should explore MSK1 activation, downstream IEGs (*Arc, c-Fos, Egr1*) [17], glial contributions, and circuit-level connectivity to elucidate its role and therapeutic potential in preclinical models of schizophrenia and ASD.

## Supplementary information

Supplementary Information

## Data Availability

The datasets generated and/or analysed during the current study are available from the corresponding author (R.D.) on reasonable request.
