# Supplementary material for: MSK1 mediates BDNF-dependent MeCP2-S421 phosphorylation in postnatal striatal development and psychiatric-relevant behaviours
Source: Mol Psychiatry. 2026 May 18;31(9):5445–60. doi: 10.1038/s41380-026-03630-3 (PMC13441968; doi:10.1038/s41380-026-03630-3)
Supplement: Supplementary file 1 [file 41380_2026_3630_MOESM1_ESM.docx]

**Supplementary Information**

**Supplementary Materials and Methods**

***Animals***

*Ethics and approvals.* All experiments using mice were conducted with permission from the “Consejería de Agricultura, Ganadería y Desarrollo Rural” of Castilla y León government, after approval by the ethical committee of the University of Salamanca and following the European Directive 2010/63/EU and the Spanish Royal Decree 53/2013-Law 32/2007.

*Maintenance.* Mice were maintained in a temperature and humidity-controlled (21 ± 1ºC, 50 ± 5%) specific pathogen free facility in individually ventilated cages, with a standard *ad libitum* diet and water and 12h:12h light-dark cycle (lights on from 8:00 a.m. to 8:00 p.m.). Experiments were conducted during the light phase.

***Vgat-IRES-CRE; Ai9-RCL^tdTomato^ mouse line generation***

*Vgat-IRES-CRE; Ai9-RCL^tdTomato^* mice were generated by breeding the mouse lines *Vgat-IRES-CRE* (B6J.129S6(FVB)-*Slc32a1^tm2(cre)Lowl^*^/^MwarJ; The Jackson Laboratory strain #028862; ^1^) with *Ai9-RCL^tdTomato^* (B6.Cg-Gt(ROSA)26Sor^tm9(CAG-tdTomato)Hze^/J; The Jackson Laboratory strain #007909; ^2^). As only one copy of the CRE and the Ai9 alleles is necessary for recombination and correct visualization of the reporter tdTomato protein, we used heterozygous and homozygous mice indistinctly. These animals were used as test subjects for immunohistochemistry experiments.

***Msk1^IV^ KO mouse line generation***

To generate *Msk1^IV^* KO mice, we used CRISPR/Cas9 gene edition technology. We designed two specific crRNAs flanking *Msk1* exon IV (Fig. 2; Supplementary Table 1) that were annealed to a common tracrRNA to generate the mature sgRNAs for editing. The crRNAs and tracrRNAs were produced by chemical synthesis by Integrated DNA Technologies (IDT, Coralville, IA, USA). Mature sgRNAs were complexed with *S. pyogenes* Cas9 endonuclease before microinjecting the ribonucleoprotein complex into C57BL/6J zygotes. These zygotes were kept at 37ºC and 5% CO_2_ in KSOM media until the following day, when 15 to 20 viable 2-cell embryos were transferred to pseudo-pregnant CD-1 female mice. The offspring was characterized by PCR, and the promising amplicons were analysed by Sanger sequencing (Fig. 2; Supplementary Table 2; *Msk1*-FW-Seq and *Msk1*-RV-Seq primers). Founders were bred with C57BL/6J mice and heterozygous offspring was back-crossed to C57BL/6J mice for four generations to establish the mouse *Msk1^IV^* KO (B6-*Rps6ka5*^em3SAL^) line. Offspring genotype was characterized by a double-PCR to detect the presence of the mutant allele and the absence of *Msk1* exon IV (Supplementary Table 2; 102/103 and 114/115 primer pairs).

***Histology***

Mice were deeply anesthetized with sodium pentobarbital (60 mg/kg body weight, intraperitoneal) and transcardially perfused, first with ice-cold PBS, followed by an equal volume of ice-cold 4% paraformaldehyde (PFA) solution in PBS. Brains were extracted and post-fixed in 4% PFA at 4°C for 3 hours, then immersed in 30% sucrose in PBS at 4°C for 48 hours for cryoprotection.

E14.5 embryos were extracted and fixated for 24h in 4% PFA at 4ºC. Then, heads were sequentially cryoprotected in crescent concentrations of sucrose solutions (10% / 20% / 30%) and sectioned on the same day.

Coronal brain sections (40 µm thick) were cut frozen on a sliding microtome equipped with a freezing device (ThermoScientific, Waltham, MA, USA, Microm HM 430) and collected sequentially.

*Immunofluorescence.* Free-floating brain slices were washed three times with PBS and permeabilized for 10 minutes with 0.25% Triton X-100 in PBS. Sections were blocked for 2 hours at room temperature in a blocking solution (0.3% Triton X-100, 10% normal donkey serum (Jackson ImmunoResearch, West Grove, PA, 017-000-121), and 5% bovine serum albumin (BSA; NZYtech, Lisboa, Portugal, MB04602) in PBS) and then incubated for 24 hours at 4°C with primary antibodies, pre-diluted in antibody solution (0.3% Triton X-100, 5% normal donkey serum, 1% BSA in PBS). The following primary antibodies were used (Supplementary Table 5): rabbit monoclonal anti-MSK1 (C27B2, Cell Signalling (Danvers, MA, USA), 1:1500) and rabbit monoclonal anti-DARPP-32 (Santa Cruz Biotechnology, Dallas, TX, USA, 271111, 1:500). After incubation, tissue samples were washed four times with PBS at room temperature (15 minutes each) and then incubated for 1 hour in the dark with secondary antibodies and 4 μM Hoechst 33258 (Invitrogen Waltham, MA, USA, H1399) previously diluted in antibody solution. The following secondary antibodies (Supplementary Table 5) were used: donkey anti-rabbit Alexa 488 (ThermoFisher, 21206, 1:400) and donkey anti-mouse Alexa 555 (ThermoFisher, 31750, 1:400). Following three 10-minute PBS rinses, brain sections were mounted onto Superfrost glass slides pre-coated with gelatine solution (50 mM Tris-HCl pH 7.5, 0.2% gelatine from porcine skin; Sigma-Aldrich (Saint Louis, MO, USA), G1890) and air-dried at room temperature in the dark. Slides were finally coverslipped with Mowiol/DABCO mounting medium (0.1% Mowiol 4-88 (Polysciences Inc., Warrington, PA, 17951-500), 0.26% glycerol 2.5% DABCO (Acros Organics, 112470250), 0.1 M Tris-HCl pH 8.5) and stored at 4°C in the dark until imaging.

*Developmental MSK1 characterization.* Representative coronal sections (40 µm thick) of the somatosensory cortex, hippocampus and striatum from *Vgat-IRES-CRE; Ai9-RCL^tdTomato^* mice at postnatal days 5, 10, 15, and 30 (n=3 animals per age group, 3 sections per brain region of interest) were selected. These slices were immunostained with rabbit monoclonal anti-MSK1 (C27B2, Cell Signalling, 1:1500), donkey anti-rabbit Alexa 488 (ThermoFisher, 21206, 1:400) and 4 μM Hoechst 33258 as described above. High-resolution single plane mosaics (2048 × 2048-pixel resolution, 400 Hz acquisition speed) were acquired at 20× magnification using a Leica Stellaris 8 confocal microscope (Leica Microsystems, Wetzlar, Germany) with consistent laser gain, intensity, pinhole, and detection filter settings across samples. Multi-channel merged regions were imported into Arivis Vision4D software (Zeiss, Oberkochen, Germany) for 2D proper segmentation and co-labelling analysis. Nuclear Hoechst and MSK1 signals were segmented using the fluorescence-based cellpose model (deep-learning based algorithm), whereas GABAergic cell bodies (tdTomato^+^) were detected by using integrated AI with Machine Learning for segmentation. Subsequently, MSK1 and tdTomato co-labelling quantification was performed using a semi-automated pipeline based on the software Import Document Object, Segment Feature Filter, Splitting and Compartments operations. From each animal, 6 regions of interest corresponding to each brain area included in the study (both hemispheres) were outlined and analysed.

*Volumetric analysis.* Brain coronal sections were serially obtained at 40 µm thickness using a cryotome and divided into 10 consecutive series. The series containing the first coronal section of each structure (bregma level located by using Allen Brain Reference Atlas (https://atlas.brain-map.org/)) was identified and subjected to immunostaining with rabbit monoclonal anti-DARPP-32 (271111, Santa Cruz Biotechnology, 1:500) as described above. Nuclei were counterstained with 4 µM Hoechst 33258. Single-plane tile-scan 20x magnification confocal images (1024- × 1024-pixel resolution) were acquired using an inverted Leica Stellaris 8 confocal microscope (Leica Microsystems) across all sections spanning the rostro-caudal extent of cortex, hippocampus and striatum, maintaining consistent laser power, gain, and detection filter settings. Both hemispheres in each image were delineated using the Allen Brain Reference Atlas as a guide (https://atlas.brain-map.org/), and their areas were calculated with Arivis Vision4D software. The volume for each region was estimated using Cavalieri’s Principle ($Vc=d \sum_{i=1}^{n} (A_{i})$), considering the distance between each brain slice (d), the calculated area of each region in each coronal section (A_i_) and the number of sections containing the somatosensory cortex, the hippocampus or the striatum (n) respectively (n=3 animals per genotype. A series comprises between 9 and 11 sections).

***Quantitative Real-Time PCR (qPCR).***

cDNA was synthesized from 0.5 µg of total RNA, isolated from somatosensory cortex, hippocampus or striatum samples using TRI Reagent™ (ThermoFisher Scientific, Waltham, MA, USA, AM9738) according to the manufacturer’s instructions, with RevertAid reverse transcriptase (Thermo-Fisher Scientific, EP0442) following the provided protocol. Quantitative PCR (qPCR) was performed using Power SYBR Green Master Mix (Applied Biosystems, ThermoFisher Scientific, A25780) and gene-specific primers for *Msk1*, *Pgc1α,* *Gad1*, *Gad2*, *Gabrg3*, *Darpp-32*, *Gfap,* *Irak1*, *Satb1*, *Dlk1, Pvalb, Drd1*, *Drd3*, *Drd2*, *Grin1a*, *Psd95*, *Htr2c* and *Tbp* (designed using IDT tool, see Supplementary Table 6). Reactions were run on a QuantStudio 7 Real-Time PCR System (ThermoFisher Scientific) according to the manufacturer’s instructions. Relative gene expression was analysed using the 2⁻^ΔΔCt^ method, with *Tbp* as the reference gene (n=3 animals per genotype).

***Quantitative western blot.***

Total protein lysates from cell cultures were prepared using RIPA buffer (25 mM Tris-HCl pH 7.5, 150 mM NaCl, 1% Triton X-100, 1% sodium deoxycholate, 0.1% SDS supplemented with protease and phosphatase inhibitor cocktails (Pierce, ThermoFisher Scientific, A32960). Brain tissue samples mixed with Tissue-RIPA buffer (50 mM Tris-Hcl pH 7.4, 150 mM NaCl, 1mM EDTA, 1% Triton X-100, 1% sodium deoxycholate, 1% SDS, protease and phosphatase inhibitor cocktails) were sonicated at 100% amplitude, 1 cycle using a UP100H Ultrasonic Processor (Hielscher Ultrasound Technology) Protein concentrations were determined using the BCA Protein Assay Kit (Pierce, ThermoFisher Scientific, A65453). Equal amounts of protein (25–35 µg) were separated by electrophoresis on 10% Bis-Tris polyacrylamide gels and transferred to Immobilon-FL PVDF membranes (Merck-Millipore, Darmstadt, Germany, IPFL00010) following manufacturer’s protocols and recommendations. Membranes were washed once in TBS (Tris-buffered saline), methanolized and blocked for 1 hour at room temperature in Intercept-TBS Blocking Buffer (LI-COR (Lincoln, NE, USA), 927-60001). Membranes were incubated overnight at 4°C with primary antibodies diluted in blocking buffer supplemented with 0,2% Tween-20: rabbit monoclonal anti-MSK1 (Cell Signalling, C27B2, 1:1000), mouse monoclonal anti-β-actin (Proteintech (Rosemont, IL, USA), 669-1-Ig, 1:10000), mouse monoclonal anti-phospho-pan-Trk (Cell Signalling, 9141, 1:1000), rabbit polyclonal anti-MeCP2 phospho-S421 (Abcam (Cambridge, UK), ab254050,1:500), rabbit monoclonal anti-pERK1/2 (Cell Signalling, 9101, 1:3000), rabbit monoclonal anti-phospho-CREB phospho-S133 (Cell Signalling 9198, 1:1000), rabbit polyclonal anti-MeCP2 (Proteintech, 10861-1-AP, 1:500), and rabbit polyclonal anti-MSK1 phospho-Thr581 (Cell Signalling, 9595, 1:1000). After incubation, membranes were rinsed and washed twice (20 minutes per wash) with TBS-T (1X TBS with 1% Tween-20) and then incubated with fluorescent secondary antibodies diluted in a mix of the same blocking solution + 0,2% Tween-20 + 0,01% SDS for 1 hour at room temperature: IRDye 800CW donkey anti-rabbit IgG (LI-COR, 926-32213, 1:20000) and IRDye 680LT donkey anti-mouse IgG (LI-COR, 926-68022, 1:20000). Finally, membranes were rinse and similarly washed with TBS-T solutions and washed again during 5 minutes with TBS 1X solution in order to reduce background signals. Images were captured using an Odyssey XF Imaging System (LI-COR), and densitometry was quantified with Image Studio 6.0 Software (LI-COR).

***Golgi-Cox staining, neuronal morphological and dendritic spine quantification.***

WT and *Msk1*^IV^ KO mice were euthanized by cervical dislocation, and brains were rapidly dissected and immersed in Golgi-Cox solution, prepared as previously described ^3^, and incubated in the dark at room temperature for 14 days to allow impregnation of neuronal structures, with the solution replaced after the first 48 hours to ensure optimal staining. Following impregnation, brains were transferred to a 30% sucrose solution in PBS for 5 days at 4°C for cryoprotection. 200 µm-thick coronal sections (as recommended for neuronal arborization visualization) were collected using the microtome referred above and immersed in 6% sucrose solution. Sections were mounted onto gelatine-coated slides and air-dried. For the staining, sections were briefly rinsed in distilled water, followed by incubation in 15% ammonium hydroxide for 10 minutes in the dark, fixation in Kodak Rapid Fixer for 10 minutes and rinsing in distilled water. Finally, sections were dehydrated by sequentially rinsing them through a graded ethanol series (50%, 70%, 95%, 100%), cleared in xylene, and coverslipped with Entellan™ mounting medium (107961; Sigma-Aldrich).

Brightfield images of Golgi-Cox-stained neurons were acquired using a Zeiss Axio Observer microscope either with a 20x/0.8 NA dry objective for neuronal morphology reconstruction or a 100x/1.4 NA oil-immersion objective for dendritic spine quantification. Z-stack images were captured at 0.5 µm intervals to ensure detailed visualization of neuronal architecture and spines morphology. For the analysis of neuronal morphology, the Zeiss Arivis Vision4D software was utilized, employing its Neuron Tracing Tool to have semi-automatic 3D reconstructions of neurons from the z-stack images. From these reconstructions, dendritic length, branching complexity, and arborization patterns were measured. All analyses were conducted blind to experimental conditions to eliminate bias, with a minimum of 10 neurons per animal analysed, 3 animals per genotype, to ensure robust statistical representation. For dendritic spine analysis, 3 wild-type and *Msk1*^IV^ KO animals were used, analysing 15 medium spiny neurons’ secondary dendrite segments per animal, using RECONSTRUCT software (University of Texas, <https://synapseweb.clm.utexas.edu/software>) to quantify the number of dendritic spines and classify them according to their morphological parameters, as previously described ^4^.

**Medium spiny neuron spontaneous activity recordings**

8- to 12-week-old mice were anaesthetized with pentobarbital and transcardially perfused with ice-cold N-methyl-D-glucamine (NMDG)-based solution saturated with 95% O₂ / 5% CO₂ (in mM): 93 NMDG, 2.5 KCl, 1.2 NaH₂PO₄, 30 NaHCO₃, 20 HEPES, 2 thiourea, 3 sodium pyruvate, 10 MgSO₄·7H₂O, 0.5 CaCl₂·2H₂O, 25 D-glucose, and 5 sodium ascorbate (pH 7.3 adjusted with 10 N HCl; osmolarity 300–310 mOsm/L). To obtain and maintain acute brain slices, we followed an adapted method to preserve brain slices from adult and aging animals ^5^. In brief, the brain was quickly removed and placed in ice-cold NMDG solution saturated with 95% O₂ / 5% CO₂. Brains were sectioned into 325 µm-thick coronal slices using a VT-1200S vibratome (Leica Microsystems) and incubated for 10 min in a NaCl-HEPES solution (in mM): 92 NaCl, 2.5 KCl, 1.2 NaH₂PO₄, 30 NaHCO₃, 20 HEPES, 2 thiourea, 3 sodium pyruvate, 2 MgSO₄·7H₂O, 2 CaCl₂·2H₂O (pH = 7.3 with NaOH or HCl; osmolarity 275–280 mOsm/L) at 32°C. Then, slices were stored for at least 1 h in a submerged holding chamber at room temperature (25–28°C) in artificial cerebrospinal fluid (aCSF) containing (in mM): 126 NaCl, 3 KCl, 1.25 NaH₂PO₄, 2 MgSO₄·7H₂O, 26 NaHCO₃, 10 D-glucose, 2 CaCl₂·2H₂O, and 0.4 ascorbic acid (pH = 7.4 adjusted with NaOH or KCl). Striatal slices were transferred to a recording chamber, continuously superfused with oxygenated aCSF at a rate of 2 mL/min using a peristaltic pump (Gilson). Striatal neurons were visualized using infrared gradient contrast video microscopy (E600FN, Eclipse Workstation, Nikon) and a water immersion objective (Nikon Fluor 40×/1.0 NA). Medium spiny neurons (MSNs) were distinguished from interneurons by their morphology. Somatic patch-clamp recordings were obtained at 28°C from individual MSNs of the dorsal striatum using patch pipettes pulled from thick-walled borosilicate glass capillaries (Sutter Instruments) on a micropipette puller (P-97, Sutter Instruments). Patch pipettes were filled with internal solution containing (in mM): 135 cesium methanesulfonate (CH_3_CsO_3_S), 5 KCl, 0.1 EGTA, 10 HEPES, 2 NaCl, 5 ATP-Mg, 0.4 GTP-Na, 10 Na₂-phosphocreatine (pH=7.3 adjusted with CsOH; osmolarity 280–290 mOsm/L). Electrodes filled with this solution had resistances of ~5–8 MΩ. Whole-cell patch recordings were obtained in voltage-clamp mode with a PC-ONE amplifier (Dagan Corporation), low-pass filtered at 1 kHz, digitized at 20 kHz (Digidata 1322A, Molecular Devices), and acquired with Clampex 10 (Molecular Devices). Series and input resistances were monitored throughout the experiment using a –5 mV pulse. Recordings were considered stable when series and input resistances did not change >20%. Cells that did not meet these criteria were discarded. Spontaneous excitatory currents (sEPSCs) were recorded at a holding potential of –70 mV (close to the chloride equilibrium potential) and spontaneous inhibitory currents (sIPSCs) at 0 mV (reversal potential of glutamatergic events). Spontaneous events were analysed using Mini Analysis (version 6.0.7, Synaptosoft). In a subset of cells, a 300 ms, –60 pA square pulse was applied at –70 mV in current-clamp configuration to estimate membrane capacitance (Cm=membrane time constant / input resistance).

***In vitro* neuronal morphology analysis.**

*Plasmid generation.* All plasmids used in this study were derived from the previously described dual-promoter lentiviral vector pLL-hSyn1-DsRED-hSyn1-EGFP ^6^. The reporter plasmid pLL-hSyn1-DsRED was generated by excising the EGFP cassette through co-digestion with NheI and EcoRI-HF restriction enzymes (New England Biolabs, Ipswich, MA, USA, R3131 and R3101S), followed by religation of the vector backbone. To construct the pLL-hSyn1-Msk1-myc-hSyn1-DsRED plasmid, the open reading frame (ORF) of *Msk1* was amplified by PCR from cDNA synthesized from total RNA extracted from the brain of a C57BL/6J mouse. Amplification was performed using the primers Msk1-NheI-FW and Msk1-EcoRI-myc-RV, and the resulting PCR product was cloned into the pLL-hSyn1-DsRED vector in place of the EGFP cassette, incorporating a myc tag at the C-terminus of *Msk1* CDS.

To generate microRNAs (miRNAs) targeting *Msk1*, sequences were designed using the BLOCK-iT™ RNAi Designer software (Invitrogen). The corresponding oligonucleotides (listed in Supplementary Table 3) were synthesized and cloned into the pcDNA6.2-GW/miR plasmid according to the manufacturer’s instructions. Individual miRNA sequences (shown in Supplementary Fig. 5B) were subsequently subcloned together into the pcDNA6.2-GW/miR vector using BglII and XhoI restriction enzymes (New England Biolabs, R0144S and ER0695). The resulting miRNA cassette (miR-1-4) was excised with BamHI-HF and NotI restriction enzymes (New England Biolabs, R3136S and R3189S) and cloned into the pLL-hSyn1-DsRED-hSyn1-EGFP plasmid, replacing the DsRED cassette. This generated the pLL-hSyn1-miRNA1-4-Msk1-hSyn1-EGFP plasmid, which enables cell-autonomous silencing of Msk1. A control miRNA plasmid, pLL-hSyn1-miRNA-Control-hSyn1-EGFP, was constructed following the same procedure, using a non-targeting miRNA sequence.

To generate the pLL-hSyn1-miRNA1-5-*Mecp2*-hSyn1-EGFP plasmid, we designed 5 miRNAs targeting *Mecp2* using the BLOCK-iT™ RNAi Designer software (Invitrogen). Sequences were synthesized and pre-cloned inside a pMK-RQ plasmid via GeneArt Custom Gene Synthesis platform (ThermoFisher). The miRNA cassette was excised with BamHI-HF and XhoI restriction enzymes in substitution of the miR-Control cassette from the pLL-hSyn1-miRNA-Control-hSyn1-EGFP vector.

All plasmid constructs were verified by PCR, Sanger sequencing, and restriction enzyme digestion prior to their use in experiments.

*Primary cultures.* Cortices and striata were dissected from embryonic day 14.5 to 16.5 (E14.5-E16.5) mouse embryos in ice-cold BG-PBS, (PBS supplemented with 0.5% D-glucose, 0.1% bovine serum albumin (BSA, NZYtech, MB04602), 10 µg/mL penicillin (ThermoFisher, J63032), and 10 µg/mL streptomycin (ThermoFisher, 455340250)). Tissues were dissociated by incubation in Trypsin-EDTA solution (Gibco, Waltham, MA, USA, 25200-056) at 37°C for 30 minutes, followed by mechanical disaggregation using a 10 mL plastic pipette. The dissociated tissue was then diluted 1:2 with supplemented Neurobasal medium (Gibco; Cat No 11570556) containing 0.2 mM L-glutamine (Gibco, 25030-081), NCS21 (Capricorn, Düsseldorf, Germany, NCS21-A), 10 µg/mL penicillin, 10 µg/mL streptomycin, soybean trypsin inhibitor (Sigma-Aldrich, 65035-M), and DNase I (Sigma-Aldrich, 11284932001). The suspension was gently disaggregated with a 10 mL pipette, filtered through a 40 µm cell strainer, and centrifuged at 800 g for 5 minutes at room temperature. The resulting cell pellet was resuspended in supplemented Neurobasal medium, and cells were plated at a density of 300000 cells/cm² onto sterile 24-well plates (ThermoScientific, 930186) containing round-glass coverslips pretreated with 1 M HNO₃ for 24 h, washed thoroughly with sterile water for cell culture (Capricorn, WAT-1000ML), and coated overnight with 100 µg/mL Poly-D-lysine (70000–150000 MW; Sigma-Aldrich, A-003-M) and 4 µg/mL laminin (Corning, New York, USA, 354232).

*Transfection, treatment, and fixation.* After 2 days *in vitro* (2 DIV), cortical and striatal neurons were transfected with 1 µg of the corresponding plasmid using Lipofectamine 2000 (Invitrogen; 11668030) at a 1:3 DNA (µg)-to-transfection reagent (µL) ratio. Transfections were performed in OptiMEM I medium (Gibco; 31985070) for 2 hours and 15 minutes at 37°C / 5% CO_2_, after which the medium was replaced with fresh supplemented Neurobasal medium as described above. At 4 DIV, recombinant brain-derived neurotrophic factor (BDNF; 50 ng/mL; PeproTech, Rocky Hill, CT, USA, 450-02-10UG) was added to the designated cultures, and at 7 DIV cells were fixed for 15 minutes at room temperature with a 4% paraformaldehyde and 4% sucrose solution. Following fixation, wells were washed twice with PBS and stored at 4°C under humidified conditions until further processing.

*Imaging and quantification.* Fixed cells on coverslips were washed twice with PBS for 5 minutes each. Nuclei were stained by incubating coverslips in a Hoechst 33258 solution (1 µg/mL in PBS; Sigma-Aldrich, 94403) for 15 minutes at room temperature, followed by two additional 5-minute washes in PBS. Coverslips were then mounted onto glass slides using Mowiol/DABCO mounting medium. Three-dimensional (3D) images spanning the full extent of transfected neurons were acquired using a Leica Stellaris SP8 confocal microscope with a 20× dry objective (NA 0.75). Acquisition parameters were kept consistent across samples expressing the same reporter protein (EGFP or DsRED) to ensure comparable fluorescence intensity and resolution. Images were imported into Arivis Vision4D software for 3D segmentation and morphometric analysis. The neuronal soma was segmented using a dedicated fluorescence-based machine learning model. Subsequently, total neurite reconstruction was performed using a semi-automated pipeline based on the software’s neuron tracing algorithm, initiated from the soma segmentation. Quantitative data, including soma size, neurite length and arbour complexity, were derived from the combined soma and neurite segmentation results.

***MSK1-MeCP2 signalling***

*Ex vivo corticostriatal stimulation*. Horizontal corticostriatal slices (350 µm thick) were prepared from P60 wild-type or *Msk1*^IV^ KO mice using a Microm HM 650 V vibratome in ice-cold (≤8°C) artificial cerebrospinal fluid (aCSF) containing (in mM): 124 NaCl, 3 KCl, 1.25 KH₂PO₄, 3.4 CaCl₂, 2.5 MgSO₄, 26 NaHCO₃, and 10 D-glucose (pH 7.3 when oxygenated with 95% O₂/5% CO₂), as previously described ^7^. Slices were transferred to a submerged recovery chamber containing oxygenated aCSF at 28–32°C and allowed to recover for 30 min. To induce physiological BDNF/TrkB signalling activation, slices were then incubated for 10 min in oxygenated modified aCSF (0 mM Mg²⁺, 5 mM Ca²⁺) supplemented with the GABA_A receptor antagonist bicuculline (100 µM; Tocris, Cat. No. 2503). Slices were subsequently maintained for an additional 50 min in standard oxygenated aCSF containing bicuculline. In parallel, to block BDNF signalling, slices were incubated for 10 min in modified aCSF supplemented with bicuculline and the BDNF scavenger TrkB-Fc (2 µg/mL; R&D Systems) and then maintained for 50 min in standard aCSF containing bicuculline and TrkB-Fc ^8^ . Control slices were maintained in standard oxygenated aCSF (without supplements) for the equivalent total duration (60 min). At the end of the incubation period, all slices were transferred to ice-cold aCSF, the striatum was rapidly dissected under a stereomicroscope, flash-frozen in liquid N₂, and stored at –80°C until processing for Western blot analysis.

*Primary cultures and treatments.* Wild-type and *Msk1*^IV^ KO striatal primary cultures were obtained from E14.5-E16.5 embryos as indicated above. Cells were plated in supplemented neurobasal media at a density of 300,000 cells/cm^2^ onto sterile 12-well plates coated with Poly-D-lysine (100 µg/mL; 70000-150000 MW; Sigma-Aldrich) and Laminin (4 µg/mL; Corning). At 14 DIV, cultures were pre-treated with U0126 (100 nM; 30 minutes; Tocris Bio-Techne R&D Systems, 1144/5), KN92 (10 nM; 1 hour; Tocris Bio-Techne R&D Systems, 4130/1) or KN93 (10 nM; 1 hour; Tocris Bio-Techne R&D Systems, 1278/1) before treatment with BDNF (50 ng/ml). After treatment, plates were washed twice with ice-cold D-PBS (Corning, 21-031-CV) and stored at -80ºC. Total protein lysates were obtained, and western blot was performed as described above (n=3-4 different cultures, each condition comprised of 3 wells from the same culture).

***MSK1-MeCP2 Interaction***

*In vivo endogenous co-immunoprecipitation.* P60 wild-type mice were sacrificed by cervical dislocation, and brains were rapidly dissected to obtain striatal samples. Tissue was sliced with a razor blade on an ice-cold metal block, transferred to a 1.5 mL Potter-Elvehjem glass tube, and homogenized in 1 mL of STM-250 buffer (250 mM sucrose, 50 mM Tris-HCl pH 8.0, 5 mM MgCl₂, 0.5% NP-40; Alfa Aesar, Waltham, MA, USA, Cat. No. J61055-AP) supplemented with complete protease and phosphatase inhibitor cocktails (Pierce, Thermo Fisher Scientific). The suspension was transferred to a 1.5 mL Protein LoBind tube (Eppendorf, Hamburg, Germany, Cat. No. 0030108116), incubated on ice for 10 min, and centrifuged at 800 g for 10 min at 4°C. The pellet was washed with 1 mL of supplemented STM-250 buffer, and the incubation and centrifugation steps were repeated. The pellet was then resuspended in 300 µL of nuclear lysis buffer (20 mM Tris-HCl pH 8.0, 1.5 mM MgCl₂, 150 mM NaCl, 0.5 mM DTT, 1% NP-40, supplemented with complete protease and phosphatase inhibitor cocktails) containing 250 U of Pierce Universal Nuclease (ThermoFisher Scientific, Cat. No. 88702). After rotation at 4°C for 1 h, the lysate was centrifuged at 17,000 g for 15 min at 4°C, and the supernatant (nuclear fraction) was transferred to a clean 1.5 mL Protein LoBind tube. For co-immunoprecipitation, 500 µg of nuclear protein lysate was incubated with 12 µg of mouse anti-MSK1 antibody (Developmental Studies Hybridoma Bank, PCRP-RPS6KA5-1A8) or mouse IgG isotype control (Invitrogen, Cat. No. 02-6502) for 1 h with rotation at 4°C. Subsequently, 50 µL of protein G magnetic beads (MedChemExpress, Cat. No. HY-K0204) were added, and incubation continued for 48 h under the same conditions. Magnetic beads were isolated using a DynaMag Spin magnet (ThermoFisher Scientific, Cat. No. 12320D), washed five times with nuclear lysis buffer, and eluted with 50 µL of 2× LDS sample buffer (ThermoFisher Scientific, Cat. No. NP0007) containing 100 mM DTT by heating at 72°C for 10 min. Eluted samples were diluted 1:1 with ultrapure water. Both eluted samples and input nuclear lysates were analysed by Western blot for MSK1 and MeCP2.

*HEK293-FT cultures*. Cells were cultured in Dulbecco’s Modified Eagle Medium (DMEM; Gibco, 21699035) supplemented with 10% heat-inactivated fetal bovine serum (FBS, Capricorn, FBS-12A), 2 mM glutamine (Gibco, 21051-024 100g), 10 mM HEPES (Biowest, Nuaillé, France, MS00X11009), 10 µg/mL penicillin, 10 µg/mL streptomycin, 1X MEM non-essential amino acids (Gibco, 11140-035), and 0.001% 2-mercaptoethanol. Cells were maintained at 37°C in a humidified incubator (Fisherbrand Waltham, MA, USA, 50153430) with 5% CO₂ and 95% humidity. Cultures were routinely tested for mycoplasma contamination using the PCR Mycoplasma Detection Set (TaKaRa, San Jose, CA, #6601).

*Overexpression of MSK1-myc and MeCP2-HA.*

HEK293-FT cells were seeded at a density of 75,000 cells/cm² in 6-well plates (ThermoScientific, 130184). The following day, cells were transfected with pLL-hSyn1-MSK1-myc-hSyn1-DsRED (see *Plasmid generation* section above) and/or pLL-hSyn1-MeCP2-HA-hSyn1-EGFP, constructed as previously described ^9^. Cell medium was changed about 30 minutes before the experiment. For transfection, 4 µg of plasmid DNA per well (4 µg per plasmid or 2 µg of each one in case of co-transfection condition) were thoroughly mixed with polyethyleneimine (PEI; 1 mg/mL, Polysciences Inc., 19850-100) at a 1:3 DNA (µg)-to-PEI (µL) ratio in DMEM, incubated for 15 minutes at room temperature, and added to the culture medium. Twenty-four hours post-transfection, cell medium was replaced with fresh supplemented DMEM, and cells were incubated for an additional 48 hours. Where indicated, phorbol 12-myristate 13-acetate (PMA; 200 nM; MedChemExpress, Monmouth Junction, NJ, USA HY-18739) was added for 1 hour prior to cell collection.

*Purification of nuclear protein lysates.*

Cells were harvested in ice-cold D-PBS (Corning, 21-031-CV), centrifuged at 800 g for 5 minutes at 4°C, and resuspended in 10 volumes of supplemented 250-STM buffer. After a 10-minute incubation on ice, the suspension was centrifuged at 800 g for 10 minutes at 4°C. The resulting pellet was resuspended in 1 volume of non-denaturing Nuclear Lysis Buffer (20 mM Tris-HCl pH 7.5, 300 mM KCl, 0.2 mM EDTA pH 8.0, 0.1% NP-40, 20% glycerol), supplemented with the same protease and phosphatase inhibitor cocktails and incubated on ice for 30 minutes. Lysates were then centrifuged at 12,000 g for 30 minutes at 4°C, and the supernatant containing nuclear proteins was collected and stored at -20°C.

*Co-Immunoprecipitation assays.*

MeCP2-HA was immunoprecipitated from nuclear protein lysates of HEK293-FT cells transfected with pLL-hSyn1-MSK1-myc and/or pLL-hSyn1-MeCP2-HA plasmids. Anti-HA magnetic beads (Sigma-Aldrich, SAE0197) were used according to the manufacturer’s instructions. A 1% aliquot of the total input lysate volume was analysed by western blot using antibodies against phosphorylated MSK1 (pMSK1) and phosphorylated MeCP2 (pMeCP2) to confirm their presence and activation state. Immunoprecipitated proteins were eluted and analysed by western blot with antibodies against MSK1 and MeCP2 (Supplementary Table 5) to assess their physical interaction.

***Behavioural Tests***

All behavioural tests were conducted at the animal facility of the Institute of Neurosciences of Castilla y León (INCyL) between 10:00 p.m. and 15:00 p.m., in a room maintained at stable temperature, humidity, and light intensity, shielded from external noise. Prior to each test, mice (WT and *Msk1^IV^* KO subjects) were acclimated for 30 minutes in individual home cages. Unless indicated, behavioural tests were performed under normal light conditions (800 Lux). The number of animals tested varied between 9 and 13 per sex and genotype.

*Open field test.*

The open field test (OF) was used to assess anxiety-like behaviours, exploratory activity, and locomotion. The arena, constructed from white, fully opaque acrylic glass, had a rectangular base (50 cm × 40 cm) and 30 cm-high walls to prevent escape. Light intensity in the room was set to 18 lux and evenly distributed in the box to achieve uniform illumination to assure that no dark and bright areas were created in the field. Mice were placed in the centre of the arena and allowed to explore freely for 10 minutes. Distance travelled (cm), time spent (s), and number of entries into the centre and peripheral zones were recorded and analysed using ANY-maze tracking software (Stoelting Co., Wood Dale, IL, USA).

*Elevated plus maze.*

Alike O.F. test, this test is used for measure anxiety-like behaviours, exploratory activity and locomotion. The arena constructed from black, fully opaque acrylic glass has a cross base and it is 42,5 cm above the floor, with two arms fully open (30 cm-long) and the other two restricted with walls (30 cm-long and 15 cm-high walls). Light intensity in the room was evenly distributed in the box to achieve uniform illumination to assure that no dark and bright areas were created in the field. For 10 minutes animal move is recorded and time spent in each arm (s) is considered in this analysis, using ANY-maze tracking software to measure it.

*Rotarod test.*

Motor coordination and balance were evaluated using a Rotarod LE 8300 (LSI Letica, Rochester Hills, MI, USA). Mice were trained over two consecutive days prior to testing. On the test day, the Rotarod was programmed to accelerate from 4 rpm to 40 rpm over 5 minutes. Each mouse underwent three trials, with a 10-minute rest interval between trials. Latency to fall (s) and maximum velocity reached (rpm) were recorded and subsequently analysed.

*Three-chamber social test.*

This test assessed social approach and interaction preferences. The arena, made of white, fully opaque acrylic glass, consisted of three chambers: a central chamber (initial placement, empty), a left chamber (containing an unknown same-sex conspecific inside a wire cage), and a right chamber (containing a novel object, an orange Lego piece, inside a wire cage). Mice were first habituated for 10 minutes in the empty arena, with free access to all chambers. For the test, mice were placed in the central chamber and allowed to explore all chambers for 10 minutes. Interaction time (s) was measured as the duration spent sniffing the cages containing the conspecific or object. Unlike studies focusing on social novelty, our analysis targeted differences in exploration and interaction between the conspecific and the object, as both stimuli were novel. Trials were recorded and analysed using ANY-maze software.

*Marble burying test.*

The marble burying test (MBT) evaluated exploratory behaviour and anxiety-like symptoms. Standard home cages were filled with 5 cm of sawdust, and 20 white glass marbles were evenly arranged on the surface in four rows of five. Mice were allowed 30 minutes to interact with the marbles. The number of marbles buried (defined as those with at least two-thirds of their surface covered by wood chips) was counted.

*Nesting test.*

This test assessed the ability of mice to construct a nest for sleeping, hibernation, maternal behaviour or reproduction. A cotton square (5.1–5.2 g) was placed in the upper left corner of a home cage containing 0.5 cm of sawdust. During the test, mice were kept individually caged at standard *ad libitum* diet and water and a 12/12h light / dark cycle. Nest quality was scored at 4 hours and 24 hours post-placement using the criteria outlined by ^10^.

*Forced swimming test.*

This test measured depression-like behaviour. During the test, mice were individually placed in a 5 L glass beaker filled with 2.5 L of fresh filtered tap water (25 ± 1°C) and allowed to swim for 7 minutes. Water was replaced between trials. Tests were video-recorded, and freezing time (s) (defined as immobility except for minimal movements to maintain flotation) was quantified as an indicator of depressive-like behaviour. The first 2 minutes were excluded from analysis to account for initial acclimation.

***Statistics and Graphical Design***

Statistical analyses and graphs were generated using GraphPad Prism 8 (Dotmatics, Boston, MA, USA). The statistical tests applied to each experiment are detailed in the respective figure legends. Illustrations were created using Inkscape (Boston, A, USA).

**Supplementary Tables**

***Supplementary Table 1.*** crRNAs used to generate Msk1*^IV^* KO mice.

| *Msk1*-crRNA1 | 5’-GGGAGGGTTTGGTCGTCATGTGG-3’ |
| --- | --- |
| *Msk1*-crRNA2 | 5’-AATCCCAGTACTTCATTAGGGGG-3’ |

**Supplementary Table 2.** Sequencing and genotyping primers for Msk1^IV^ KO mice.

| *Msk1*-FW-Seq | 5’-CAGCCATTGTTGCATGGAAC-3’ |
| --- | --- |
| *Msk1*-RV-Seq | 5’-TACTGTACAGCATGGGCTTC-3’ |
| Primer 102 | 5’-CGTCATGTGGCTGATGTTTG-3’ |
| Primer 103 | 5’-CATGGAGCAAGTCACAATCAAG-3’ |
| Primer 114 | 5’-ATGGTTAGCCCAGAGTGATTAAA-3’ |
| Primer 115 | 5’-CATTCATAAGCCCAAGGCAAC-3’ |

**Supplementary Table 3.** Single-stranded DNA oligonucleotides used to generate miRNA plasmids. These contain the flanking sequences of miRNA-155, the specific miRNA coding sequences against Msk1 (underlined) and spacer sequences.

| miR*Msk1*-1-top | 5’-TGCTGAGAATATGCTCTTTCAGTTT  CGTTTTGGCCACTGACTGACGAAACTGAGAGCATATTCT-3’ |
| --- | --- |
| miR*Msk1*-1-bottom | 5’-CCTGAGAATATGCTCTCAGTTTCGTCAGTC  AGTGGCCAAAACGAAACTGAAAGAGCATATTCTC-3’ |
| miR*Msk1*-2-top | 5’-TGCTGTACATTAGAACACCTAAGCTC  GTTTTGGCCACTGACTGACGAGCTTAGGTTCTAATGTA-3’ |
| miR*Msk1*-2-bottom | 5’-CCTGTACATTAGAACCTAAGCTCGTCA  GTCAGTGGCCAAAACGAGCTTAGGTGTTCTAATGTAC-3’ |
| miR*Msk1*-3-top | 5’-TGCTGTGAAGAGGATCTATAACAGCT  GTTTTGGCCACTGACTGACAGCTGTTAGATCCTCTTCA-3’ |
| miR*Msk1*-3-bottom | 5’-CCTGTGAAGAGGATCTAACAGCTGT  CAGTCAGTGGCCAAAACAGCTGTTATAGATCCTCTTCAC-3’ |
| miR*Msk1*-4-top | 5’-TGCTGAAAGGTTGCTTTCACACAGGT  GTTTTGGCCACTGACTGACACCTGTGTAAGCAACCTTT-3’ |
| miR*Msk1*-4-bottom | 5’-CCTGAAAGGTTGCTTACACAGGTGTCA  GTCAGTGGCCAAAACACCTGTGTGAAAGCAACCTTTC-3’ |

**Supplementary Table 4.** Primers used to generate the pLL-hSyn1-Msk1-myc-hSyn1-DsRED plasmid.

| Msk1-NheI-FW | 5’-gctagcGAGGGTGAAGATGGAGGG-3’ |
| --- | --- |
| Msk1-EcoRI-*myc*-RV | 5’-CgaattcTCA*cagatcctcttctgagatgagtttttgttc*  GTCTGAGAACTGGAAGAGGGTGTC-3’ |

***Supplementary Table 5****: Antibodies used in the study (IF: Immunofluorescence; WB: Western Blot; IP: Immunoprecipitation).*

| **Primary Antibodies** | **Company & Catalogue Nº** | **Dilution used** |
| --- | --- | --- |
| MSK1 (C27B2) Rabbit IgG | CellSignaling Cat. Nº 3489 | IF: 1:1500; WB: 1:1000 |
| MSK1 (1A8) Mouse IgG | DSHB PCRP-RPS6KA5-1A8 | IP: 24 µg / mg lysate |
| pMSK1-pThr581 Rabbit IgG | CellSignaling Cat. Nº 9595 | WB: 1:1000 |
| NeuN (A60) Mouse IgG1 | MERCK Cat Nº MAB377 | IF: 1:1000 |
| GFAP (G-A-5) Mouse IgG | Sigma-Aldrich Cat Nº G6171 | IF: 1:5000 |
| DARPP-32 (H-3) Mouse IgG1 | Santa Cruz Biotechnology  Cat Nº sc-271111 | IF: 1:1000 |
| pan-pTrk-pTyr490 Rabbit IgG | CellSignaling Cat. Nº 9141 | WB: 1:1000 |
| pERK1/2 (pThr202/pTyr204) Rabbit IgG | CellSignaling Cat. Nº 9101 | WB: 1:3000 |
| pCREB (87G3; pSer133) Rabbit IgG | CellSignaling Cat. Nº 9198 | WB: 1:1000 |
| MeCP2 Rabbit IgG | Proteintech  Cat Nº 10861-1-AP | WB: 1:500 |
| pMeCP2-pSer421 Rabbit IgG | Abcam Cat Nº ab254050 | WB: 1:500 |
| pCaMKIIα-pThr286  Mouse IgG1 | ThermoFisher Cat Nº ma1-047 | WB: 1:2000 |
| β-actin Mouse IgG2b | Proteintech Cat Nº 66009-1-Ig | WB: 1:20000 |
| **Secondary Antibodies** | **Company & Catalogue Nº** | **Dilution used** |
| Donkey anti-Rabbit IgG  Alexa Fluor™ 488 | Invitrogen Cat Nº A-21206 | IF: 1:400 |
| Donkey anti Mouse IgG  Alexa Fluor ™ 555 | Invitrogen Cat Nº A-31570 | IF: 1:400 |
| Donkey anti Mouse IgG  Alexa Fluor ™ 647 | Invitrogen Cat Nº A-31571 | IF: 1:400 |
| IRDye® 800 CW Donkey anti-Rabbit IgG 800 CW | LI-COR Cat Nº 925-32213 | WB: 1:20000 |
| IRDye® 800 CW Donkey anti-Mouse IgG 720 CW | LI-COR Cat Nº 926-68072 | WB: 1:20000 |

***Supplementary Table 6:*** *Primers used for qPCR assays.*

| **Gene** | **Forward (5’ to 3’)** | **Reverse (5’ to 3’)** |
| --- | --- | --- |
| *5-Htr2c* | GTCCCTAGCCATTGCTGATATG | AGCACATCTAGTGAAATCCAGAC |
| *Darpp-32* | GGTCTCAGAGCACTCCTCACCAG | TGCTGCACAGCTTTCAGTGAT |
| *Dlk1* | TGTCAATGGAGTCTGCAAGG | ATTCGTACTGGCCTTTCTCC |
| *Drd1* | ATGATCGTCACTTACACCAGTATC | TTCGACAGGGTTTCCATTACC |
| *Drd2* | ACATCTTTGTCACTCTGGATGTC | CCCAGACAATGGCGATCAT |
| *Drd3* | TTATCCACATCCCTGAAGCTG | ACAGTGGGTATTAAGAACGTGAG |
| *Gabrg3* | TTCCTGATCGAATAAGCCTTCAA | CACAGGTGTCCTCAAATTCCT |
| *Gad65* | GTCTGCTTCTGGTTTGTACCT | GTGGTCCCATACTCCATCATTC |
| *Gad67* | AGCCTGAGCACACAAATG | TGACTCCATCATCAGAGCTT |
| *Gfap* | CACCTACAGGAAATTGCTGGAGG | CCACGATGTTCCTCTTGAGGTG |
| *Grin1a* | TGGAGAAGCACAATTATGAGAGT | CCAGATCGCACTTCTGTGAA |
| *Irak1* | GTCTTGGATAGCCTGCAACTG | TGAGGGATTTGTCAGAGTGAAC |
| *Msk1* | CAATGAATGGCTTCAGGATG | AAAGGCATGAAAGGTTGCTT |
| *Pgc1a* | GAAATTGAGGAATGCACCGTAAA | CGACCTGCGTAAAGTATATCCA |
| *Psd95* | ACTGCATCCTTGATGTCTCAG | TCCTCTGTGATCCGCTTATTG |
| *Pvalb* | GGACATCAAGAAGGCGATAGG | GGAACACCTTCTTCACCTCAT |
| *Satb1* | TCTAGGAAGAGGAAGGCTTGG | CGCAGAAAACTGGTAACATGG |
| *Tbp* | CACCAATGACTCCTATGACCC | CAAGTTTACAGCCAAGATTCACG |

**Supplementary Figures and Legends**

**Supplementary Figure 1. Characterization of non-GABAergic MSK1-expressing cells in the somatosensory cortex at P30**

(A) MSK1 expression in cortical neurons of *Vgat-IRES-Cre; Ai9-RCL^tdTomato^* mice at postnatal day 30 (P30). A1: representative confocal tile scan (20x) of a coronal cortical section showing MSK1 (greyscale), tdTomato (orange, vast majority of GABAergic neurons), and NeuN (purple) fluorescence, with zoom insets highlighting MSK1^+^ cells (arrowheads: tdTom^-^/NeuN^-^/MSK1^+^, grey; tdTom^+^/NeuN^+^/MSK1^+^, yellow). A2: schematic dot map indicating MSK1^+^ cell subtypes (grey: non-neuronal; purple: non-GABAergic neurons; yellow: GABAergic NeuN^+^ neurons; salmon pink: GABAergic NeuN^-^ neurons). A3: quantification of GABAergic MSK1^+^ neurons as a percentage of total MSK1^+^ cells at P5 and P30, schematic of analysed somatosensory cortex (blue), and relative frequency of MSK1^+^ cell phenotypes. (B) MSK1 expression in cortical astrocytes at P30, showing a confocal tile scan (20x) of a coronal cortical section showing Hoechst (blue, nuclei), MSK1 (green), tdTomato (red, vast majority of GABAergic neurons), and GFAP (magenta, astrocytes) fluorescence. Zoom insets (40x oil immersion objective) highlight MSK1^-^ astrocytes (white arrowheads) and MSK1^+^ GFAP^-^ cells (green arrowheads). Scale bars vary by image. n=3 animals, 3 sections per animal. Data are presented as mean ± SEM, with each dot representing one animal. Statistical analysis: two-tailed unpaired Student’s t-test (***p* < 0.01).

**Supplementary Figure 2. Developmental dynamics of MSK1 expression in cortex and hippocampus**

Analysis of MSK1 expression in *Vgat-IRES-Cre; Ai9-RCL^tdTomato^* mice at postnatal days 5, 10, 15, and 30 (P5–P30) in somatosensory cortex (A) and hippocampus (B). Each block includes: (1) Representative single-plane confocal images of coronal sections showing MSK1 (greyscale), tdTomato (red, GABAergic neurons), and Hoechst (blue, nuclei) fluorescence, with hippocampal subregion zoom insets highlighting intense MSK1^+^ GABAergic neurons; (2) Schematic dot maps of MSK1^+^ non-GABAergic (grey) and GABAergic (salmon pink) nuclei; (3) Quantification of MSK1^+^ cells as a percentage of total cells and GABAergic MSK1^+^ cells as a percentage of total MSK1*^+^* cells; (4) Subregion-specific quantification of MSK1^+^ cells, GABAergic MSK1^+^ cells, and MSK1^+^ cell distribution (n=3 animals per stage, 3 sections per region). Scale bars vary by image. Data are presented as mean ± SEM, with each dot representing one animal. Statistical analysis: one-way ANOVA with Tukey’s *post hoc* test (**p* < 0.05, ***p* < 0.01, ****p* < 0.001 vs. P5). Abbreviations: P5–P30, postnatal days 5–30; L1–L6, cortical layers 1–6; CA1–CA3, *Cornua Ammonis* regions 1–3; DG, dentate gyrus.

**Supplementary Figure 3. Developmental dynamics of MSK1 expression in the striatum**

Analysis of MSK1 expression in *Vgat-IRES-Cre; Ai9-RCL-^tdTomato^* mice at postnatal days 5, 10, 15, and 30 (P5–P30) in the striatum. (1) Representative single-plane confocal images of coronal sections showing MSK1 (greyscale), tdTomato (red, vast majority of GABAergic neurons), and Hoechst (blue, nuclei) fluorescence, with zoom insets and arrowheads indicating MSK1^+^ non-GABAergic (greyscale) and GABAergic (salmon pink) nuclei. (2) Schematic dot maps of MSK1^+^ non-GABAergic (grey) and GABAergic (salmon pink) nuclei. (3) Quantification of MSK1^+^ cells as a percentage of total cells and GABAergic MSK1^+^ cells as a percentage of total MSK1^+^ cells across time points. (4) Subregion-specific quantification of MSK1^+^ cells (top), MSK1^+^ cells distribution (middle) and GABAergic and MSK1^+^ cells (bottom) (n=3 animals per stage, 3 sections per animal). Scale bars vary by image. Data are presented as mean ± SEM, with each dot representing one animal. Statistical analysis: one-way ANOVA with Tukey’s *post hoc* test was applied to panel 4 lower graphs (Total MSK1^+^ cells (%)). Abbreviations: P5–P30, postnatal days 5–30; CP, caudate-putamen; NAc, nucleus accumbens.

**Supplementary Figure 4. Region-specific *Msk1* expression levels across development**

(A) qPCR analysis of *Msk1* mRNA levels in cortex, hippocampus, and striatum at postnatal days 7 (P7) and 21 (P21), normalised to *Tbp* using the 2^⁻ΔΔCt^ method (a.u. arbitrary units). (B) Western blot and quantification of MSK1 protein levels in cortex, hippocampus, and striatum at P7 and P21 relative to beta-actin levels and normalized as % to P21 striatal levels. Data are presented as mean ± SEM (n=3 animals per group). Statistical analysis: one-way ANOVA with Tukey’s *post hoc* test (**p* < 0.05, ****p* < 0.001, comparisons between regions at the same age; ^#^*p* < 0.05, ^##^*p* < 0.01 ^###^*p* < 0.001, comparisons between ages at the same region). (C) Heatmap of *Msk1* expression (FPKM) in cortical neurons at early postnatal stages, based on published RNAseq data ^11^. (D) Heatmap of *Msk1* expression (log_2_ TPM) in striatal cells from 5–7-week-old male mice, based on published scRNAseq data sheet ^12^.

**Supplementary Figure 5. Validation of *Msk1^IV^* KO and region-specific volume analysis**

(A) Upper panel: Schematic representation of murine WT MSK1 structure (863 aminoacids). The phosphorylation sites necessary for MSK1 activation have been highlighted, distinguishing between ERK1/2 or p38 mediated phosphorylation (orange), C-terminal kinase domain autophosphorylation (blue) and N-terminal kinase domain autophosphorylation (grey) sites. Activating phosphorylation sites are previously described in ^13,14^ and human-murine equivalence has been established using Mass-Spectrometry annotated data from PhosphoSite ([phosphosite.org](http://phosphosite.org)). Lower panel: Schematic representation of the predicted truncated peptide resulting from deletion of *Msk1* exon IV in mice (136 aminoacids). This peptide would contain the initial part of the N-terminal kinase domain without its activating phosphorylation site (Ser212), plus 6 additional amino acids derived from the Open Reading Frame (ORF) shifting derived from exon IV elimination.

(B) Coronal sections (Bregma ≈ +0.045 mm) from P60 wild-type (WT) and *Msk1^IV^* KO mice, immunostained DARPP-32 (red), MSK1 (green) and counterstained with Hoechst. (C–E) Representative coronal sections of WT and *Msk1^IV^* KO mice, immunostained for DARPP-32 and counterstained with Hoechst, used for volumetric analysis of somatosensory cortex, hippocampus, and striatum via Cavalieri’s principle, referencing the Allen Brain Atlas. Scale bar: 500 µm (C, D) 1 mm (B, E). Cell number quantification for each region was determined from Hoechst staining using CellPose integrated module in Arivis Vision4D and normalised to the estimated volume. n=3 animals per genotype, 9 to 11 sections analysed per animal. Data are presented as mean ± SEM (n=3 animals per group). Statistical analysis: two-tailed unpaired Student’s t-test (**p* < 0.05). Abbreviations: STR, striatum; LV, lateral ventricle; LS, lateral septum; SI, substantia innominata; EP, endopiriform cortex; HY, hypothalamus; PIR, piriform cortex; cc, corpus callosum; int, internal capsule; GP, globus pallidus; AMY, amygdala.

**Supplementary Figure 6. BDNF-dependent MeCP2 Ser421 phosphorylation is mediated by the ERK1/2–MSK1 pathway in striatal neurons**

(A) Quantitative Western blot analysis of striatal lysates from P60 WT (upper panel) and *Msk1^IV^* KO (lower panel) *ex vivo* stimulated slices, assessing pMeCP2-S421, pERK1/2, and pCREB-Ser133 levels relative to actin in control, stimulated, or stimulated in the presence of TrkB-Fc (2 µg/mL; WT only). n=3, each n represents a pool of 3–4 brain slices from the same mouse. Data are presented as mean ± SEM. Statistical analysis: one-way ANOVA with Dunnett’s post hoc test versus control condition for WT data; two-tailed unpaired Student’s t-test for *Msk1^IV^* KO data (*p < 0.05). (B-C) Quantitative Western blot analysis of total lysates from E14.5–E16.5 + 14 DIV WT and *Msk1^IV^* KO cultured striatal neurons, assessing pTrkB, pERK1/2, and pMeCP2-Ser421 levels relative to actin after pretreatment with MEK/ERK inhibitor U0126 (100 nM, 30 min), inactive CaMKII inhibitor KN92 (10 µM, 1 h), or active CaMKII inhibitor KN93 (10 µM, 1 h), followed by BDNF stimulation (50 ng/mL, 30 min or 1 h). n=3–4, each n consisting of a pool of 3 wells from the same culture. Data are presented as mean ± SEM. Statistical analysis: two-tailed unpaired Student’s t-test (**p* < 0.05, ***p* < 0.01). Right panel: Western blot against pCaMKIIα in WT striatal neuron lysates to confirm KN93 inhibition. (D) Schematic of the signalling pathway mediating BDNF-dependent MeCP2 phosphorylation in striatal neurons.

**Supplementary Figure 7. Embryonic expression of MSK1 in GABAergic neurons and validation of knockdown constructs for *in vitro* morphology experiments**

(A) Immunofluorescence detection of MSK1 (white) in E14.5 *Vgat-IRES-Cre; Ai9-RCL-^tdTomato^* embryos (tdTomato in red, labelling the vast majority of GABAergic neurons), showing MSK1 presence in the cortical plate and ganglionic eminence/striatal primordium in both GABAergic (salmon pink arrowheads) and non-GABAergic (grey arrowheads) cells. Scale bar: 500 µm (main images); 50 µm (insets). (B–D) Validation of knockdown constructs. Wild-type E14.5–E16.5 + 7 Days *in vitro* (DIV7) primary neurons were transfected with (B) pLL-hSyn1-miRNA-Control-hSyn1-EGFP (control miRNA), (C) pLL-hSyn1-miRNA1-4-*Msk1*-hSyn1-EGFP (*Msk1*-targeting miRNAs; cassette encodes four concatenated pri-pre-miRNAs targeting *Msk1* mRNA, validated by Western blot in HEK293-FT cells co-transfected with MSK1-myc), or (D) pLL-hSyn1-miRNA1-5-*Mecp2*-hSyn1-EGFP (*Mecp2*-targeting miRNAs; cassette encodes five concatenated pri-pre-miRNAs targeting *Mecp2* mRNA, validated by Western blot in HEK293-FT cells co-transfected with HA-MeCP2). (E) *Msk1^IV^* KO E14.5–E16.5 + 7 DIV primary neurons transfected with pLL-hSyn1-DsRED (reporter) or pLL-hSyn1-MSK1-myc-hSyn1-DsRED (MSK1 rescue). Panels B–E also show representative 3D confocal projections and Arivis Vision4D reconstructions of 7 DIV striatal neurons from E14.5–E16.5 WT and *Msk1^IV^* KO embryos, transfected at 2 DIV, treated with BDNF (50 ng/mL) at 4 DIV, fixed at 7 DIV, and imaged (scale bar: 100 µm).

**Supplementary Figure 8. Global MSK1 deficiency disrupts BDNF-dependent cortical neuron morphology in vitro**

Cultured cortical neurons from E14.5–E16.5 wild-type (WT) and *Msk1^IV^* KO embryos were transfected at 2 days *in vitro* (DIV) with plasmids under the human synapsin 1 promoter (hSyn1). WT neurons were transfected with hSyn1-miR-Ctrl-EGFP (control miRNA), hSyn1-miR-*Msk1*-EGFP (*Msk1*-targeting miRNAs), or hSyn1-miR-*Mecp2*-EGFP (*Mecp2*-targeting miRNAs). *Msk1*^IV^ KO neurons were transfected with hSyn1-DsRED (reporter) or hSyn1-MSK1-DsRED (MSK1 rescue). At DIV 4, cultures were treated with BDNF (50 ng/mL), fixed at DIV 7, and imaged using a Leica Stellaris SP8 confocal microscope. Morphological analysis was performed with Arivis Vision4D. (A) Representative 3D reconstructions of analysed cortical neurons (scale bar: 150 µm). (B) Quantification of soma volume. (C) Quantification of average neurite length and number of primary neurites. (D–E) Quantification of arbour complexity: average section length, branching points per neurite, average terminal section length, and terminal points per neurite. Cell-autonomous effects were assessed by intra-genotype one-way ANOVA with Dunnett’s *post hoc* test versus basal condition (WT + miR-Ctrl: ****p* < 0.001; *Msk1*^IV^ KO: ^ǂǂ^*p* < 0.01, ^ǂǂǂ^*p* < 0.001). Cell-context effects were analysed by two-tailed unpaired Student’s t-test (WT + miR-Ctrl vs. *Msk1*^IV^ KO, WT + miR-Ctrl + BDNF vs. *Msk1*^IV^ KO + BDNF, WT + miR-Ctrl vs. *Msk1*^IV^ KO + Rescue: ^#^*p* < 0.05, ^###^*p* < 0.001). Data are presented as mean ± SEM. n=15–29 neurons per condition from 3 different neuronal cultures.

**Supplementary References**

1.	Vong L, Ye C, Yang Z, Choi B, Chua S, Lowell BB. Leptin Action on GABAergic Neurons Prevents Obesity and Reduces Inhibitory Tone to POMC Neurons. *Neuron*. 2011;71(1):142-154. doi: 10.1016/j.neuron.2011.05.028

2.	Madisen L, Zwingman TA, Sunkin SM, et al. A robust and high-throughput Cre reporting and characterization system for the whole mouse brain. *Nat Neurosci*. 2010;13(1):133-140. doi:10.1038/nn.2467

3.	Zhong F, Liu L, Wei JL, Dai RP. Step by Step Golgi-Cox Staining for Cryosection. *Front Neuroanat*. 2019;13:62. doi:10.3389/fnana.2019.00062

4.	Risher WC, Ustunkaya T, Singh Alvarado J, Eroglu C. Rapid Golgi Analysis Method for Efficient and Unbiased Classification of Dendritic Spines. Dunaevsky A, ed. *PLoS ONE*. 2014;9(9):e107591. doi:10.1371/journal.pone.0107591

5.	Ting JT, Daigle TL, Chen Q, Feng G. Acute Brain Slice Methods for Adult and Aging Animals: Application of Targeted Patch Clamp Analysis and Optogenetics. In: Martina M, Taverna S, eds. *Patch-Clamp Methods and Protocols*. Vol 1183. Methods in Molecular Biology. Springer New York; 2014:221-242. doi:10.1007/978-1-4939-1096-0_14

6.	Gascón S, Paez-Gomez JA, Díaz-Guerra M, Scheiffele P, Scholl FG. Dual-promoter lentiviral vectors for constitutive and regulated gene expression in neurons. *J Neurosci Methods*. 2008;168(1):104-112. doi:10.1016/j.jneumeth.2007.09.023

7.	Jia Y, Gall CM, Lynch G. Presynaptic BDNF Promotes Postsynaptic Long-Term Potentiation in the Dorsal Striatum. *J Neurosci*. 2010;30(43):14440-14445. doi:10.1523/JNEUROSCI.3310-10.2010

8.	Rex CS, Lin CY, Kramár EA, Chen LY, Gall CM, Lynch G. Brain-Derived Neurotrophic Factor Promotes Long-Term Potentiation-Related Cytoskeletal Changes in Adult Hippocampus. *J Neurosci*. 2007;27(11):3017-3029. doi:10.1523/JNEUROSCI.4037-06.2007

9.	Yazdani M, Deogracias R, Guy J, Poot RA, Bird A, Barde YA. Disease Modeling Using Embryonic Stem Cells: MeCP2 Regulates Nuclear Size and RNA Synthesis in Neurons. *Stem Cells*. 2012;30(10):2128-2139. doi:10.1002/stem.1180

10.	Lewarch CL, Hoekstra HE. The evolution of nesting behaviour in Peromyscus mice. *Anim Behav*. 2018;139:103-115. doi:10.1016/j.anbehav.2018.03.008

11.	Favuzzi E, Deogracias R, Marques-Smith A, et al. Distinct molecular programs regulate synapse specificity in cortical inhibitory circuits. *Science*. 2019;363(6425):413-417. doi:10.1126/science.aau8977

12.	Gokce O, Stanley GM, Treutlein B, et al. Cellular Taxonomy of the Mouse Striatum as Revealed by Single-Cell RNA-Seq. *Cell Rep*. 2016;16(4):1126-1137. doi:10.1016/j.celrep.2016.06.059

13.	Arthur JSC. MSK activation and physiological roles. *Front Biosci J Virtual Libr*. 2008;13:5866-5879. doi:10.2741/3122

14.	Cargnello M, Roux PP. Activation and Function of the MAPKs and Their Substrates, the MAPK-Activated Protein Kinases. *Microbiol Mol Biol Rev*. 2011;75(1):50-83. doi:10.1128/MMBR.00031-10
